# Cost-effectiveness of point-of-care versus centralised, laboratory-based nucleic acid testing for diagnosis of HIV in infants: a systematic review of modelling studies

**DOI:** 10.1016/S2352-3018(23)00029-2

**Published:** 2023-05-04

**Authors:** Stanzi M le Roux, Jasantha Odayar, Catherine G Sutcliffe, Phillip P Salvatore, Gatien de Broucker, David Dowdy, Nicole C McCann, Simone C Frank, Andrea L Ciaranello, Landon Myer, Lara Vojnov

**Affiliations:** a Division of Epidemiology & Biostatistics, School of Public Health, University of Cape Town, Cape Town, South Africa; b Department of Epidemiology, Johns Hopkins Bloomberg School of Public Health, Baltimore, MD, USA; c Department of International Health, Johns Hopkins Bloomberg School of Public Health, Baltimore, MD, USA; d Medical Practice Evaluation Center, Department of Medicine, Massachusetts General Hospital Boston, MA, USA; e Division of Infectious Diseases, Massachusetts General Hospital Boston, MA, USA; f Global HIV, Hepatitis and STI Programme, World Health Organization, Geneva, Switzerland

## Abstract

**Background:**

Point-of-care (POC) nucleic acid testing for diagnosis of HIV in infants facilitates earlier initiation of antiretroviral therapy (ART) than with centralised (standard-of-care, SOC) testing, but can be more expensive. We evaluated cost-effectiveness data from mathematical models comparing POC with SOC to provide global policy guidance.

**Methods:**

In this systematic review of modelling studies, we searched PubMed, MEDLINE, Embase, the National Health Service Economic Evaluation Database, Econlit, and conference abstracts, combining terms for “HIV” + “infant”/”early infant diagnosis” + “point-of-care” + “cost-effectiveness” + “mathematical models”, without restrictions from database inception to July 15, 2022. We selected reports of mathematical cost-effectiveness models comparing POC with SOC for HIV diagnosis in infants younger than 18 months. Titles and abstracts were independently reviewed, with full-text review for qualifying articles. We extracted data on health and economic outcomes and incremental cost-effectiveness ratios (ICERs) for narrative synthesis. The primary outcomes of interest were ICERs (comparing POC with SOC) for ART initiation and survival of children living with HIV.

**Findings:**

Our search identified 75 records through database search. 13 duplicates were excluded, leaving 62 non-duplicate articles. 57 records were excluded and five were reviewed in full text. One article was excluded as it was not a modelling study, and four qualifying studies were included in the review. These four reports were from two mathematical models from two independent modelling groups. Two reports used the Johns Hopkins model to compare POC with SOC for repeat early infant diagnosis testing in the first 6 months in sub-Saharan Africa (first report, simulation of 25 000 children) and Zambia (second report, simulation of 7500 children). In the base scenario, POC versus SOC increased probability of ART initiation within 60 days of testing from 19% to 82% (ICER per additional ART initiation range US$430–1097; 9-month cost horizon) in the first report; and from 28% to 81% in the second ($23–1609, 5-year cost horizon). Two reports compared POC with SOC for testing at 6 weeks in Zimbabwe using the Cost-Effectiveness of Preventing AIDS Complications-Paediatric model (simulation of 30 million children; lifetime horizon). POC increased life expectancy and was considered cost-effective compared with SOC (ICER $711–850 per year of life saved in HIV-exposed children). Results were robust throughout sensitivity and scenario analyses. In most scenarios, platform cost-sharing (co-use with other programmes) resulted in POC being cost-saving compared with SOC.

**Interpretation:**

Four reports from two different models suggest that POC is a cost-effective and potentially cost-saving strategy for upscaling of early infant testing compared with SOC.

**Funding:**

Bill & Melinda Gates Foundation, Unitaid, National Institute of Allergy and Infectious Diseases, National Institute of Child Health and Human Development, WHO, and Massachusetts General Hospital Research Scholars

## Introduction

Early initiation of antiretroviral therapy (ART) substantially improves the survival and health of children living with HIV.^1^ Without ART, a third of children living with HIV do not survive the first year of life.^2^ ART for symptomatic infants reduces infant mortality, but benefits vary by timing of initiation.^3^ In 2007, a landmark trial compared same-day initiation to deferred initiation (based on clinical parameters) of treatment in asymptomatic infants (median age 7 weeks).^1^ Within 9 months of follow-up, the early treatment group had a 76% survival benefit, despite 66% of infants in the deferred initiation group having initiated ART following clinical deterioration. A stark mortality difference was already detectable within the first 3 months; two in three deaths occurred within 6 months of follow-up, demonstrating the relative urgency of ART in infants.^1^ Accordingly, WHO advocates universal ART for children living with HIV, with a strong recommendation for rapid initiation.^4^ However, early treatment initiation requires early diagnosis, which in turn requires testing access with prompt turnaround times for, and clinically appropriate management responses to, positive results. As HIV infection in children younger than 18 months (hereafter referred to as infants) can only be reliably diagnosed using molecular testing (nucleic acid testing, NAT), implementation strategies to achieve early diagnosis have been hampered by logistical and cost constraints in most high burden settings.^5^, ^6^ In 2020, a third of infants born to women living with HIV received no HIV testing within the recommended first 2 months of life globally, while 75% did not receive timely testing in west and central Africa.^7^ In addition, 52% of all children younger than 15 years living with HIV remained undiagnosed.^7^ However, new technological advances have enabled point-of-care (POC) NAT-based testing, allowing decentralised diagnosis by trained non-laboratory staff and facilitating same-day results with rapid ART initiation for children living with HIV.^4^, ^5^, ^8^ POC testing has the potential to revolutionise infant diagnosis from logistic and cost perspectives, enabling rapid scale-up for early testing and ART initiation. A strong evidence base supports accuracy, feasibility, and acceptability of POC infant diagnosis.^9^, ^10^, ^11^, ^12^ Mathematical modelling studies enable broader understanding and evaluation of the cost-effectiveness and implications of health-care interventions, under different clinical and contextual scenarios and using a range of anticipated settings and costs.^13^ However, modelling studies have inherent limitations, predominantly related to quality of the data used in the model, model structure, and implicit assumptions.^14^ Synthesis of findings from different cost-effectiveness models can boost understanding of the broader applicability of findings, to provide guidance for optimal resource allocation in the context of paediatric HIV.^15^

**Research in context**

**Evidence before this study**

Point-of-care (POC) nucleic acid testing (NAT) for infant diagnosis of HIV has the potential to greatly improve progress to elimination of paediatric HIV and AIDS. Compared with the current standard of care (SOC; centralised laboratory-based NAT), POC-NAT for infant diagnosis showed high diagnostic accuracy and substantial clinical benefit in several systematic reviews and meta-analyses. However, with budgetary constraints, programmatic concerns around implementation of POC-NAT for infant diagnosis centre on the cost-effectiveness and affordability of POC compared with SOC. Mathematical modelling of cost-effectiveness enables contextualisation of both costs and effectiveness under various assumptions and different, simulated situations. We did a systematic literature review of published and unpublished studies reporting cost-effectiveness of POC compared with SOC-NAT for infant HIV diagnosis using mathematical models. We searched PubMed, MEDLINE, Embase, the NHS Economic Evaluation Database, Econlit, and conference abstracts, combining terms for “HIV” + “infant”/”early infant diagnosis” + “point-of-care” + “cost-effectiveness” + “mathematical models”, without restrictions from database inception to July 15, 2022. We extracted data on health and economic outcomes from the identified original studies, and incremental cost-effectiveness ratios (ICERs) for synthesis. Study quality was assessed using the Consolidated Health Economic Evaluation Reporting Standards checklist. Model focus, approaches, and assumptions differed widely, resulting in a range of ICER estimates, with degree of cost-effectiveness varying by publication. To provide policy guidance regarding the cost-effectiveness of POC versus SOC NAT for infant HIV diagnosis, we provide a narrative synthesis of all cost-effectiveness modelling data currently available.

**Added value of this study**

The robustness of data from individual modelling studies is directly influenced by model structure, assumptions, and input data. Synthesis of data from different cost-effectiveness models can partly address some of these shortcomings, boosting understanding of the broader applicability of findings, and provide guidance for optimal resource allocation in the context of paediatric HIV. Our structured summary of model results, similarities, and differences provides policy makers with a single overview alongside additional insight that seeks to translate knowledge to action.

**Implications of all the available evidence**

Despite differences in model type, setting, and time horizon, all four modelling reports included in this synthesis presented convincing evidence of the cost-effectiveness of POC-NAT testing over SOC-NAT testing in sub-Saharan African settings. The cost-effectiveness for POC over SOC was primarily driven by improved linkage to HIV care and treatment (a result of faster turnaround time and lower risk of loss to follow-up under POC), resulting in improved survival for children living with HIV across models. Taken together with the substantial evidence for accuracy, effectiveness, feasibility, and acceptability, this structured synthesis of cost-effectiveness modelling data supports urgent investment in and scale-up of POC-NAT to increase access to HIV infant diagnostics across high burden settings, ideally with a shared platform use approach.

We aimed to systematically assess reports of cost-effectiveness models comparing POC-NAT with centralised, laboratory-based NAT (standard of care, SOC) for infant HIV diagnosis (<18 months).

## Methods

### Search strategy and selection criteria

Following a previously developed protocol (appendix pp 1–4) we used iterative search strategies in PubMed, MEDLINE, Embase, National Health Service Economic Evaluation Database (University of York Centre for Reviews and Dissemination), Econlit, and conference abstracts. We adjusted search strategies by database, combining terms for “HIV” + “infant”/”early infant diagnosis” + “point-of-care” + “cost-effectiveness” + “mathematical models”, without restrictions (last full search Feb 26, 2020; updated search July 15, 2022; appendix p 5). Eligibility was assessed using a population, intervention, comparison, outcomes, and study (PICOS) framework (population [HIV-exposed children younger than 18 months], intervention [POC-NAT for primary diagnosis of HIV], comparator [laboratory-based NAT for primary diagnosis of HIV], outcome [cost-effectiveness], study design [mathematical modelling]). The primary outcomes of interest were incremental cost-effectiveness ratios (ICERs, comparing POC with SOC) for ART initiation and survival of children living with HIV. Two reviewers (SMlR and JO) reviewed titles and abstracts independently, with full-text review for any article potentially addressing the PICOS question. Disagreements were resolved through discussion. Studies not addressing the full PICOS framework were excluded.

### Quality assessment, data extraction, and analysis

We assessed methodological quality using the Consolidated Health Economic Evaluation Reporting Standards statement (appendix pp 6–11).^16^ We extracted data on geographical setting, model type, population, perspective, time horizon, and discounting; model assumptions and parameters, and scenario and sensitivity analyses; platforms and testing algorithms; overall health outcomes and economic outcomes; and relative cost-effectiveness, measured in ICERs. All costs were extracted as per the publication, and, where relevant, adjusted to 2018 US$ using the Consumer Price Index Inflation calculator. ICERs were reported as per the index publication. The Preferred Reporting Items for Systematic Reviews and Meta-analyses checklist for this research is included in the appendix (pp 12–13). Data were synthesised qualitatively, in recognition of the substantial heterogeneity of the available mathematical models.

### Role of the funding source

The funders had no role in study design or conduct; the collection, analysis, or interpretation of the data; or the preparation, review, or approval of the manuscript. The corresponding author had full access to all the data in the study and had final responsibility for the decision to submit for publication.

## Results

Our search identified 75 records including 13 duplicates. 57 of 62 non-duplicate articles were excluded and five were reviewed in full text. One article was excluded as it was not a modelling study, and four qualifying studies were included in the review (figure, appendix p 14).^17^, ^18^, ^19^, ^20^, ^21^ These four reports were from two mathematical models from two independent modelling groups (table 1). The Cost-Effectiveness of Preventing AIDS Complications-Paediatric (CEPAC-P) model was used to evaluate clinical benefits and cost-effectiveness of POC versus SOC at 6 weeks of age^18^ and cost-effectiveness of POC versus strengthened laboratory-based testing (S-SOC) versus SOC, at age 6 weeks;^19^ both reports focused on Zimbabwe. The Johns Hopkins University (JHU) model assessed cost-effectiveness of POC versus SOC for a representative setting in sub-Saharan Africa, with testing at age 6 weeks and 9 months;^20^ and cost-effectiveness of POC versus SOC in Zambia, with testing at birth and age 6 weeks and 6 months.^21^ All reports were considered high quality. Model parameters are shown in table 2, with additional details in the appendix (pp 15–36).

**Figure:**
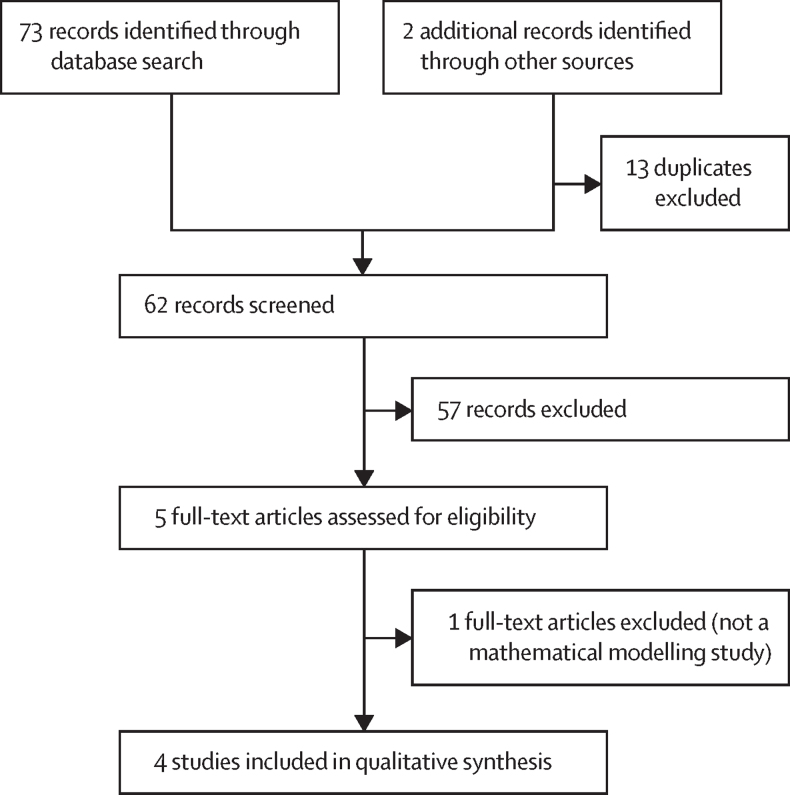
Study selection

**Table 1:** Overview of similarities and differences in model setting, type, and approaches

	**CEPAC model 1 (Zimbabwe)**	**CEPAC model 2 (Zimbabwe)**	**JHU model 1 (sub-Saharan Africa)**	**JHU model 2 (Zambia)**
**Publication details**				
Title	Clinical effect and cost-effectiveness of incorporation of point-of-care assays into early infant HIV diagnosis programmes in Zimbabwe: a modelling study.^18^	Strengthening existing laboratory-based systems vs investing in point of care assays for early infant diagnosis of HIV: a model-based cost-effectiveness analysis.^19^	Modelling the cost-effectiveness of point-of-care platforms for infant diagnosis of HIV in sub-Saharan African countries.^20^	The cost-effectiveness of scaling up rapid point-of-care testing for early infant diagnosis of HIV in southern Zambia.^21^
Primary author and affiliation	Simone C Frank, Massachusetts General Hospital, Boston, MA USA	Nicole C McCann, Massachusetts General Hospital, Boston, MA USA	Phillip P Salvatore, Johns Hopkins Bloomberg School of Public Health, Baltimore, MD USA	Gatien De Broucker, Johns Hopkins Bloomberg School of Public Health, Baltimore, MD USA
Journal	*Lancet HIV*	*JAIDS*	*AIDS*	*PLoS ONE*
Year	2019	2020	2021	2021
**Setting**				
Country	Zimbabwe	Zimbabwe	“Representative high burden setting in [sub-Saharan Africa] where implementation of POC is likely to be considered”	Zambia
EID testing strategy	6 weeks	6 weeks	6 weeks and 9 months	Birth, 6 weeks, and 6 months
**Model overview**				
Type	Individual-level, state-transition, micro-simulation	Individual-level, state-transition, micro-simulation	Decision tree model	Decision tree model
Cycles	Monthly	Monthly	NA	NA
Discount	3%	3%	Undiscounted	“Based on lifespan of vehicle/platform and the time horizon (5 years)”
Perspective	Health system	Health system	Health system: EID programme specifically	Health system: EID programme specifically
Time horizon: costs	Birth to death	Birth to death; 5-year budget impact	9-month costs	5-year costs
Time horizon: clinical outcomes	Birth to death	Birth to death; 5-year budget impact	Excess mortality over 18 months among infants with HIV who went undiagnosed or initiated on ART more than 60 days after specimen collection	Excess mortality from time of HIV infection to ART start or age 12 months if ART not started
Predetermined ICER threshold	GDP per person (2016 $): ≤$1010 per YLS	Cost per YLS equivalent to providing second line ART: <$580 per YLS (2017 $); GDP per person in Zimbabwe (2017 $) $1600 per YLS	Not explicitly stated; overall GDP per person in sub-Saharan Africa at time of analysis, $1574·20; additional costs of POC over SOC expressed as % of per-person health expenditure in select countries.	Not explicitly stated; GDP per person for Zambia at time of analysis, $1510
2018 equivalent GDP per person for setting in $, using World Bank world development indicators	$1684	$1684	$1589	$1556
Primary comparison	POC *vs* SOC*; POC has 1 platform, 1 testing algorithm, 1 implementation model; hub-and-spoke model (46% spokes); dried blood spot transported within 1 h from spoke sites with same-day result	POC *vs* strengthened SOC *vs* SOC*; POC has 2 platforms, 1 testing algorithm, 1 implementation model (as model 1); strengthened SOC (laboratory): as for SOC, but with increased frequency transport, data tracking system, additional staff numbers and training, improved maintenance	POC *vs* SOC*; POC has 3 platforms, 2 testing algorithms; implementation model not clear	POC *vs* SOC*; POC has 2 platforms, 4 testing algorithms, 3 implementation models; base case implementation model: 40 POC platforms at 40 high-volume facilities; 100% testing by POC, all 7500 children annually; 61% of children testing onsite, 39% referred in person from other facilities
Primary outcome	ICER: $ per YLS	ICER: $ per YLS	% initiated on ART by 60 days; ICER: $ per additional infant initiating ART by 60 days	% initiated on ART by 60 days; ICER: $ per additional infant initiating ART by 60 days
Secondary outcomes	Survival at 12 weeks; survival at 12 months; life expectancy; lifetime costs per child	Survival at 12 months; life expectancy; lifetime costs per child; budget impact over 5 years	% initiated on ART by 18 months; excess 18-month mortality before ART initiation; ICER per additional outcome^1^, ^2^	% initiated on ART by 12 months; excess 12-month mortality before ART initiation; % children treated with ART but false HIV-positive test; ICER per additional outcome^1^, ^2^

All values are US$. CEPAC=Cost-Effectiveness of Preventing AIDS Complication-Paediatric model. JHU=Johns Hopkins University. EID=early infant diagnosis (PCR-based diagnosis in the first 9–18 months of life). YLS=years of life saved. GDP=gross domestic product. ART=antiretroviral therapy. POC=point of care. SOC=standard of care (centralised, laboratory-based testing). ICER=incremental cost-effectiveness ratio. na=not applicable.

* Centralised laboratory, peripheral sites transport dried blood spot tests for analysis; assumes platform sharing.

**Table 2:** Overview of similarities and differences in model parameters

			CEPAC model 1 (Zimbabwe)*	CEPAC model 2 (Zimbabwe)*	**JHU model 1 (sub-Saharan Africa)**†	JHU model 2 (Zambia)*
**Modelled cohort characteristics**						
Number of HIV-exposed children simulated for base case scenarios			30 million to 300 million	30 million	25 000 (2 tests per child annually, about 50 000 tests)	37 500 (annual cohort of 7500 children, over 5 years)
Mean infant age, months			0 (SD 0)	0 (SD 0)	Birth cohort	Children enter the testing cohort at different ages
Mothers with CD4 cell counts ≤350 cells per μL before ART, %			36%	36%	Not specified	Not specified
Mothers receiving ART during pregnancy and breastfeeding, %			Base case 93%	Base case 96%	High coverage 93%; low coverage 48%	Base case 93% [73–99%]; high coverage 99%; low coverage 73%
Breastfeeding, % of all mother–infant pairs			80% [0–100%]	94%	Not specified	Not specified
Mean duration of breastfeeding, months			18 (SD 2) [6–24]	17 (SD 1) [6–24]	Not specified	Not specified
Proportion tested at birth			NA	NA	Not modelled	0·40 [0·15–0·80] of all children enter the cohort at birth
Proportion tested at 6 weeks			1·0	1·0	0·85 (0·83–0·87)	0·45 [0·45–0·10] of all children enter the cohort at 6 weeks
Proportion tested at 6 or 9 months			NA	NA	0·8 (0·7–0·9) of those who tested negative at 6 weeks returning at 9 months	0·15 [0·40–0·10] of all children enter the cohort at 6 months
Proportion returning for subsequent testing			NA	NA	0·8 (0·7–0·9) of those who tested negative at 6 weeks returning at 9 months	Those returning for any repeat EID testing after birth: 0·8 [0·75–0·85] if PVT received; 0·6 [0·35–0·85] if PVT not received
**Transmission risk overview (prevalence of infant HIV infection at time of testing)**						
All transmission routes, %			5·2% (overall, model output)	3·0% (overall, model output)	Among infants first tested at 6 weeks (model input): HIV transmission risk of 2% (0·5–5%) if PVT was received, or 20% (10–30%) if no PVT received	Overall model output: 3·9%; depending on age and previous testing (model input): 1–8% if PVT received, 2–30% if PVT not received
Intra-uterine or intra-partum (once-off), %			1% if mother received ART; 17–27% if mother did not receive ART, depending on maternal CD4 cell count	0·26–1·4% if mother received, depending on when ART initiated; 18·0–19·7% if mother did not receive ART, depending on whether mother has acute or chronic HIV	NA	For testing at birth, 1% if PVT received, 8% if PVT not received; for testing at 6 weeks (following a negative birth test) 1% if PVT received, 2% if PVT not received; for testing at 6 weeks (with no prior testing), 2% if PVT received, 22% if PVT not received
Postpartum, %			Monthly, while breastfeeding: 0·19% if mother received ART, 0·24–1·28% if mother did not receive ART, depending on maternal CD4 count and exclusivity of breastfeeding	Monthly, while breastfeeding: 0·11% if mother received ART, 0·89% if mother did not receive ART	New transmissions between 6 weeks and 9 months 1·7% (0·5–3%); transmission risk at age 9 months (time of test) among infants first tested at 9 months (ie, would include all peripartum transmissions), model input: 9% transmission risk (4–14%) if PVT received, 30% (15–45%) if no PVT received	For testing at 6 months (previously tested negative at 6 weeks): 1% if PVT received, 1% if PVT not received; testing at 6 months (had negative test at 6 weeks but no birth test): 1% if PVT received, 2% if PVT not received; for testing at 6 months (had no previous testing): 8% if PVT received, 30% if PVT not received
Duration of transmission risk in model			Throughout breastfeeding (up to 2 years of age)	Throughout breastfeeding (up to 2 years of age)	Up to 9 months of age	Up to 6 months of age
**Mortality and morbidity inputs**						
HIV-positive child: clinical events (monthly, range by type of event, child age, and CD4 cell counts or percentages), %			Monthly range 0·0–11·6%	Monthly range 0·0–11·6%	NA	NA
HIV-positive child: estimated overall mortality risks (untreated HIV; range by timing of infection, age, and duration of risk)			Monthly range 0·11–20%	Monthly range 0·11–20%	Overall range 11–44%	Overall range 1·2–33%
ART efficacy (% with HIV RNA <50 copies per mL at 24 weeks on ART)			First line ART efficacy 82–91% depending on age	First line ART efficacy 82–91% [90–96%] depending on age	Not specified	Not specified
**EID cascade parameters**						
EID uptake, %			100% [30–100%] at 6 weeks	100% at 6 weeks	85% at 6 weeks	95% by 6 weeks
Probability of receiving first test results						
	SOC (laboratory)		80% [70–100%]	79%	Not explicitly modelled but reflected in probability of starting ART before 60 days	Not explicitly modelled but reflected in probability of starting ART before 60 days
	Strengthened SOC (laboratory)		NA	91% [83–98%]		
	POC (primary comparison)		99% [40–100%]	98%		
Mean delay between confirmatory test and receipt of results						
	SOC (laboratory)		2 (SD 0) months [1–4]	61 days	Not explicitly modelled but reflected in probability of starting ART before 60 days	Not explicitly modelled but reflected in probability of starting ART before 60 days
	Strengthened SOC (laboratory)		NA	53 days [5–60]		
	POC (primary comparison)		0 (SD 0) months [0–1]	1 day		
Probability of initiating ART after testing positive						
	SOC (laboratory), %		51·9% [50–100%]	52%	<60 days, 20% (13–27%); <18 months 48% (43–53%)	<60 days, 30% (13–42%); <12 months 55% (35–65%)
	Strengthened SOC (laboratory), %		NA	71% [66–86%]	NA	NA
	POC (primary comparison), %		98·5% [40–100%]	86%	<60 days, 89% (85–94%); <18 months, 95% (85–100%)	<60 days, 90%; <12 months 94%
**Assay characteristics**						
SOC assay characteristics						
	Sensitivity (all ages, for all transmission routes)		100% [90–100%]	100%	100%	100%
	Specificity (all ages)		99·6% [98·8–100%]	99·6%	100%	100%
	Error rate, %		1·4% [1·4–12%]	3·8%	Not specified	1%
POC assay characteristics						
	Sensitivity (all ages, for all transmission routes)		96·9% [60–100%]	96·9%	96% GeneXpert (both); 98% m-PIMA; 80% sensitivity analysis	96·8% [92·7–98·9%] GeneXpert; 99·0% [96·4–99·9%] m-PIMA
	Specificity (all ages)		100% [90–100%]	99·9% [90–100%]	99·8% GeneXpert (both); 99·9% m-PIMA	99·91% GeneXpert; 99·97% m-PIMA
	Error rate, %		6·0% [6–10%]	7·8%	Not specified	9% both platforms
Overview of estimated costs			In 2016 $	In 2017 $	In 2018 $	In 2018 $
	Cost per test		Incorporates capital cost	Incorporates capital cost	Recurrent cost only	Not provided per test
		SOC (laboratory)	$24·18	$18·10	$15 (14–16)	Not provided per test
		Strengthened SOC (laboratory)	NA	$30·47	NA	Not provided per test
		POC (primary comparison)	$27·61	$30·71, GeneXpert-IV; $29·22, m-PIMA	$20 (18–22), GeneXpert-IV & Edge; $25 (23–27), m-PIMA	Not provided per test
	Costs for HIV-specific clinical care					
		HIV care per month (range by age, CD5 and CD4 cell counts)	$32·75–33·69 [0·5x–3x]	$32·75–33·69 [0·5x–3x]	NA	NA
		CD4 test	$4·79	$4·79	NA	NA
		Viral load test	$17·50	$17·50	NA	NA
		ART regimen per month (range by regimen, dose, and age and weight of infant)	$8·50–44·00 [0·5x–3x]	$5·49–22·62 [0·5x–3x]	NA	NA

EID=early infant diagnosis (PCR-based diagnosis in the first 9–18 months of life). ART=antiretroviral therapy. POC=point of care. SOC=standard of care (centralised, laboratory-based testing). NA=not applicable. PVT=prevention of vertical transmission.

* Value [range examined in sensitivity analysis] or additional information.

† Value (95% uncertainty range) or additional information. All monetary values are US$. CEPAC=Cost-Effectiveness of Preventing AIDS Complications-Paediatric model. JHU=Johns Hopkins University.

There were notable similarities and differences between model assumptions and outcomes. All four reports estimated ICERs comparing POC with SOC for scale-up of testing services, in high HIV burden settings. All models assumed pre-existing SOC infant diagnosis testing systems, with peripheral clinics sending batches of dried blood spots to a centralised laboratory. Cost parameters, based on programmatic and research data, included capital and recurrent costs for scale-up and maintenance (data sources are shown in the appendix pp 15–36).^17^, ^22^, ^23^, ^24^, ^25^, ^26^, ^27^ All models assumed an integrated platform use in SOC, with costs and use shared between early infant diagnosis (EID), HIV viral load, and tuberculosis testing. For example, using data provided by the Clinton Health Access Initiative, JHU model 1 assumed 50% of SOC platforms were used for EID purposes. Across models, SOC results were returned using electronic communication tools where available, with variable delays and probability of initiating ART following a positive result; base-case POC implementation strategies relied on platform placement at high throughput sites, with either transported dried blood spots or in-person referrals from less busy sites (appendix pp 15–36). Three reports specifically assessed both Cepheid GeneXpert (primarily GeneXpert-IV) and Abbott m-PIMA as the POC platforms of interest (appendix pp 15–36).^21^, ^22^, ^23^ Allocated assay sensitivity (SOC 100% and POC 96–99%) and specificity (SOC 99·6–100%; POC, 99·8–100%) were similar across models. ICERs were calculated from a health system perspective, specifically the EID programme (all HIV-exposed children who receive testing), with primary outcomes occurring among children living with HIV. All four reports concluded that POC testing increases the probability of early ART initiation compared with SOC with clear survival benefits for children living with HIV, and acceptable incremental costs or, in some cases, cost reduction (tables 3, 4).^18^, ^19^, ^20^, ^21^ Two reports (one from each model) provided budgetary impact analyses, both with favourable conclusions.^19^, ^20^

Differences between the reports primarily reflected differences in modelling approaches, outcome definitions, modelled age(s) of testing, and implementation approaches for the upscaling of POC testing (tables 1, 2). The CEPAC model is a state-transition model with a lifetime horizon for both costs and health outcomes.^18^, ^19^ Using monthly cycles, the model has been extensively validated and calibrated for clinical variability across risks for HIV transmission, HIV disease progression, acute and chronic morbidity, and mortality.^28^ The model allows substantial variation in clinical input parameters and ART efficacy across time. Both of the CEPAC modelling analyses included in this manuscript were based on the POC implementation strategy and pilot programme assessment data from the Elizabeth Glaser Pediatric AIDS Foundation (EGPAF) and Unitaid EID initiative in Zimbabwe.^17^ Both reports focused on US$ per year of life saved (YLS), with testing at age 6 weeks, comparing POC with SOC.^17^, ^18^, ^19^ CEPAC model 2 also included a third comparison group of S-SOC, using data from a study in Kenya.^22^ This strategy included improved sample transport (daily instead of weekly as per SOC), a data tracking system with electronic alerts, additional laboratory staff and training, and improved laboratory maintenance.^22^ By comparison, the JHU model is a decision-tree model with shorter time horizons for both costs (9 months for the sub-Saharan African analysis and 5 years for the Zambian-focused analysis) and outcomes (age 18 months for sub-Saharan Africa and 12 months for Zambia).^20^, ^21^ The JHU analyses modelled a cohort of children over time, with various probabilities of leaving and re-entering care for repeat testing over time; input data were predominantly from previous EID projects.^26^, ^27^, ^29^, ^30^, ^31^ The primary outcome for both JHU models was the probability of ART initiation within 60 days in children living with HIV; additional outcomes were the probability of ever initiating ART (by 18 months in sub-Saharan Africa and 12 months in Zambia), and HIV-related deaths averted before ART initiation. The JHU modelling reports also assessed the effect on clinical outcomes and cost-effectiveness of different practical approaches to scaling up of POC, by comparing ICERs for POC versus SOC across different testing algorithms and implementation models.^20^, ^21^

The two modelling approaches aligned in overall conclusions, indicating cost-effectiveness of POC compared with SOC. In both JHU modelling analyses, a substantially greater proportion of children living with HIV was projected to initiate ART within 60 days of testing. However, as costs were spread over only 9 months compared with 5 years, the ICERs for model 1 were markedly higher than model 2. ART initiation probability increased from 19% to 82% (ICER per additional ART initiation, range $430–1097) in model 1; and from 28% to 81% in model 2 (ICER per additional ART initiation, $23–1609; table 3, appendix p 37).^20^, ^21^ In the Zambian setting (JHU model 2), the programme with integrated testing and costs (15–25% use for infant diagnosis, based on data from a platform co-utilisation feasibility study in Zimbabwe)^32^ was cost-saving (ICER of –$831 per additional ART initiation within 60 days; table 3, appendix p 37).^21^ Similar clinical benefits with differences in ICERS due to varying cost horizons were seen at later ages. By 12 months (JHU model 2) and 18 months (JHU model 1), 34–41% more children living with HIV were projected to have initiated ART through POC compared with SOC testing services, at a cost of $1467 (9-month cost horizon, JHU model 1) or $37 (5-year cost horizon, JHU model 2) per additional ART initiation. Integrated testing and costs reduced these ICERs to $406 (JHU model 1) and –$1307 (cost-saving, JHU model 2).^20^, ^21^ ICER per death averted before ART initiation was $3426 for JHU model 1 and $90 for JHU model 2. As with other outcomes, POC was cost-saving compared with SOC using integrated costing with a 5-year horizon (table 3).^20^, ^21^ Total programme costs for both SOC and POC for the JHU models are shown in table 3; detailed costing information is provided in appendix (pp 15–36). The JHU model 1 report provided estimates of budgetary impact on select countries, using population data from the World Bank and UNAIDS.^20^ At the time of analysis, estimated SOC costs represented a small percentage of total health expenditure per capita (0·06% in DR Congo, 0·04% in Kenya, and 0·21% in Malawi). Varying by platform choice and degree of use, the incremental costs of POC implementation in these countries were estimated to increase the percentage of health budget by less than 0·5% in all three countries. In the CEPAC models, POC versus SOC testing provided clinically important survival benefits, with 12 weeks’ survival increasing from 76% to 83% among children living with HIV (CEPAC model 1), and 12 months’ survival from 67–69% to 76–78% (CEPAC models 1 and 2).^18^, ^19^ In turn, POC was associated with roughly 3 years longer undiscounted life expectancy than with SOC for children living with HIV in both CEPAC models.^18^, ^19^ Although life expectancy for the full birth cohort (all HIV-exposed infants) was not substantially improved in either CEPAC model (about 0·1 year longer for POC than for SOC), the cost per YLS was within the pre-specified ICER thresholds ($711 per YLS for CEPAC model 1, $2018; and $830 per YLS for CEPAC model 2, $2018; table 4, appendix pp 15–36). ICERs from the CEPAC models were notably higher than those in the JHU models, reflecting lifetime HIV and ART treatment costs in CEPAC models, with effectiveness measured by potential clinical consequences of POC testing rather than short-term probabilities of ART initiation. In CEPAC model 2, S-SOC was associated with only slightly better 1-year survival for children living with HIV than SOC (69·9% *vs* 67·3%; undiscounted life expectancy 22·71 *vs* 21·74 years), but worse survival than POC (75·6%; life expectancy 24·49 years).^19^ Similarly, costs for S-SOC were lower than for POC but higher than for SOC. In cost-effectiveness analysis, S-SOC was weakly dominated (ie, a less efficient use of resources compared with both the other strategies, with higher ICER despite lower cost of resources). In budget impact analysis, the SOC testing approach linked 1680 children living with HIV to HIV care at a cost of $15·7 million (0·93% of the total national HIV budget of Zimbabwe in 2017). By contrast, the POC testing approach (GeneXpert-IV) linked 4480 children living with HIV to care at $23·1 million (1·37% of the total Zimbabwean HIV budget, appendix p 38).^19^

**Table 3:** Short-term and medium-term epidemiological and economic outcomes comparing POC with laboratory-based testing for early infant diagnosis of HIV

	**SOC-NAT**		POC-NAT: GeneXpert-IV*		**%POC–%SOC**	**ICER per outcome (2018 $), POC *vs* SOC**		**Gross decimal product per person in 2018 $**
	% of children with HIV	Total programme costs (2018 $)	% of children with HIV	Total programme costs (2018 $)		EID 100% costs	Integrated costs†	
**Children with HIV initiating on ART within 60 days**								
JHU model 1 (sub-Saharan Africa)‡	19%	$720 000	82%	$1 647 000	63% more children	$966	$268	$1589
JHU model 2 (Zambia)§	28%	$2 880 000	81%	$2 900 000	53% more children	$23	−$831 (cost-saving)	$1556
**Children with HIV ever initiating on ART**								
JHU model 1 (sub-Saharan Africa): within 18 months†	46%	$720 000	87%	$1 647 000	41% more children	$1467	$406	$1589
JHU model 2 (Zambia): within 12 months‡	51%	$2 880 000	85%	$2 900 000	34% more children	$37	−$1307 (cost-saving)	$1556
**HIV-related deaths among children with HIV**								
JHU model 1 (sub-Saharan Africa): within 18 months†	23%	$720 000	5%	$1 647 000	18% fewer deaths	$3426	$953	$1589
JHU model 2 (Zambia): within 12 months‡	18%	$2 880 000	4%	$2 900 000	14% fewer deaths	$90	−$3199 (cost-saving)	$1556

SOC=standard-of-care (centralised laboratory testing with dried blood spots sent in from peripheral clinics). POC=point-of-care. NAT=nucleic acid testing. ICER=incremental cost-effectiveness ratio (comparing POC with SOC). ART=antiretroviral therapy. EID=early infant diagnosis. JHU=Johns Hopkins University.

* POC with testing algorithm using POC for both primary and confirmatory test and for tie-breaker tests where required; implementation for JHU model 1 not specified, but for JHU model 2, implementation model based on 61% of clinics covered with POC on premises, and 39% peripheral (low turnover) testing clinics referred mother–infant pairs directly to clinics with on-site POC; testing costing for mPIMA not shown as more expensive with higher ICERS across outcomes; although GeneXpert Edge showed better ICERS than IV in JHU model 1, only GeneXpert-IV results shown here, to optimise model comparability.

† GeneXpert used for EID, use 15% for JHU model 1 and 10% for JHU model 2 (costs shared by tuberculosis programme and non-EID HIV programmes, for viral load testing and diagnostic purposes); assumes that EID is prioritised over viral load and tuberculosis testing.

‡ Decision-tree model with 9-month horizon for costing and 18 months for clinical outcomes, focused broadly on sub-Saharan Africa; assumes prevention of vertical transmission coverage range of 93%, 96% sensitivity of POC assay, and 20% input probability of ART initiation within 60 days.

§ Decision-tree model with 5-year horizon for costs and 12 month clinical outcomes, focused on Zambia; assumes prevention of vertical transmission coverage of 93%, 97% sensitivity of POC assay, and 30% input probability of ART initiation within 60 days.

**Table 4:** Long-term epidemiological and economic outcomes for children with HIV comparing POC with laboratory-based testing for early infant diagnosis of HIV: all results for Zimbabwe

		**SOC-NAT (costs converted to 2018 $)**			POC-NAT*(costs converted to 2018 $)			**Absolute difference in outcomes, POC–SOC**	**ICER comparing POC with SOC, 2018 $ per year of life saved**
		Outcomes	Lifetime costs per person, undiscounted	Lifetime costs per person, discounted	Outcomes	Lifetime costs per person, undiscounted	Lifetime costs per person, discounted		
**All HIV-exposed children**									
Life expectancy (undiscounted)									
	CEPAC model 1†	62·5 years	$683	..	62·6 years	$721	..	0·1 years longer	..
	CEPAC model 2‡	63·3 years	$338	..	63·4 years	$399	..	0·1 years longer	..
Life expectancy (discounted)									
	CEPAC model 1†	25·7 years	..	$387	25·8 years	..	$439	0·1 years longer	$711
	CEPAC model 2‡	26·0 years	..	$205	26·0 years	..	$246	No difference	$850
**Children with HIV**									
Survival at 12 weeks of age									
	CEPAC model 1†	76%	$12 377	..	83%	$14 083	..	7% more children	..
Survival at 12 months of age									
	CEPAC model 1†	69%	$12 377	..	78%	$14 083	..	10% more children	..
	CEPAC model 2‡	67%	$10 593	..	76%	$12 088	..	9% more children	..
Life expectancy (undiscounted)									
	CEPAC model 1†	22·7 years	$12 377	..	25·5 years	$14 083	..	3·2 years longer	..
	CEPAC model 2‡	21·7 years	$10 593	..	24·5 years	$12 088	..	2·8 years longer	..

SOC=standard of care (centralised laboratory testing with dried blood spots sent in from peripheral clinics). POC=point of care. NAT=nucleic acid testing. ICER=incremental cost-effectiveness ratio (comparing POC with SOC). ART=antiretroviral therapy. CEPAC=Cost-Effectiveness of Prevention of AIDS Complications-Paediatric model.

* POC with testing algorithm using POC for both primary and confirmatory test; implementation for model 1 not specified, but for model 2, implementation model based on hub-and-spoke model with 54% of clinics covered with POC on premises, and 46% peripheral (low turnover, spokes) testing clinics sending dried blood spots to primary testing sites (1 h delivery, same-day results assumed).

† Individual-level, state-transition model with life-time horizon for both costs and outcomes; assumes 80% breastfeeding (mean duration 18 months); prevention of vertical transmission coverage of 93%, 96% sensitivity of POC assay, and SOC turnaround time around 2 months. ICER threshold $1684 average Zimbabwe GDP per person in 2018; model 1 does not specify testing platform.

‡ Individual-level, state-transition model with lifetime horizon for both costs and outcomes; assumes 94% breastfeeding (mean duration 17 months), prevention of vertical transmission coverage of 96%, 96% sensitivity of POC assay, and SOC turnaround time around 2 months; ICER threshold $1684 average Zimbabwe GDP per person in 2018. Model 2 specifies GeneXpert-IV with gel battery; testing costing for mPIMA similar.

All four models used extensive scenario and sensitivity analyses (appendix pp 15–36); cost-effectiveness curves are shown in each of the original publications.^18^, ^19^, ^20^, ^21^ Salient results from variation in key parameters are summarised here. Both JHU model reports assessed the impact of prevention of vertical transmission (ie, maternal ART) coverage.^20^, ^21^ For sub-Saharan Africa (JHU model 1), using POC with a 9-month cost horizon and keeping all other model parameters constant, a low prevention of vertical transmission coverage of 48% resulted in lower ICER than for coverage of 93% ($417 *vs* $966 per additional ART initiation within 60 days).^20^ This result reflected a higher prevalence of infant HIV, conversely increasing the number of children living with HIV and initiating ART. In the Zambian report (JHU model 2), ICER did not vary substantially across coverage, ranging from 73% to 99%.^21^ Both CEPAC models assessed the potential impact of reduced ART effectiveness following a POC (but not SOC) HIV diagnosis. In model 1 (base-case 82–91% ART efficacy following POC, depending on age), the POC versus SOC ICER remained similar within a plausible range of ART efficacy (70–100%).^19^ POC only became less cost-effective than SOC in the unlikely scenario of post-POC testing ART efficacy below 45%. POC (*vs* SOC) also remained cost-effective when ART efficacy was reduced for both POC and SOC. ICERs remained robust through the full tested ART efficacy range in CEPAC model 2 (90–96%).^19^ The CEPAC models also assessed the effects of varying treatment costs. In model 1, POC testing was no longer cost-effective compared with SOC if HIV-related health-care costs doubled, or ART costs tripled, across both SOC and POC strategies.^18^ ICERs remained below the threshold across the examined range of treatment costs (sensitivity analysis range, 0·5-fold to three-fold increase in costs).^19^ Variations in SOC diagnostic accuracy, turnaround time, and probability of ART initiation were assessed in both CEPAC and JHU models. In the CEPAC model 1, POC remained more cost-effective than SOC across a range of plausible variations in SOC assay characteristics, turnaround time, and probability of initiating ART, in one-way, most multi-way, and seven of nine best-worst case scenario analyses (appendix pp 15–36).^18^ In all scenario analyses, POC testing resulted in greater life expectancy for children living with HIV. In the JHU models, the relative cost-effectiveness of POC decreased as the programmatic performance of SOC improved. For example, in the JHU model 1 report, increasing the probability of ART initiation within 60 days following SOC testing from 25% to 75% reduced the projected number of additional children living with HIV initiating ART after POC from 880 to 150. At base-case costing levels, POC was no longer cost-effective if more than 60% of children living with HIV initiated ART within 60 days of SOC (*vs* basecase scenario, 15–27% initiations).^20^ In the JHU model 2, ICERs remained stable when the probability of initiating ART within 60 days after SOC increased from 30% (base-case) to 43% (ICER changed from $23 to $22 per additional ART initiation). Across models, a lower POC sensitivity was associated with lower POC effectiveness and, in turn, higher ICERs. POC remained cost-effective compared with SOC within plausible POC assay sensitivity ranges in CEPAC model 1 (93·3–99%), only losing cost-effectiveness at the implausibly low sensitivity of 65%.^18^ In the JHU models, POC was potentially cost-saving even when POC sensitivity was as low as 80% (appendix pp 15–36).^21^ Different POC implementation strategies affected degree of cost-effectiveness substantially. JHU model 2 directly compared three POC implementation approaches against SOC in Zambia, assuming 100% use of the POC platform for infant HIV diagnosis.^21^ In the base-case (implementation model 1), POC platforms were placed at 40 high turnover facilities (>1·5 tests per day), covering about 60% of all HIV-exposed infants with other sites referring mother–infant pairs directly to these facilities (assumes no testing delays, and no health system costs for referral). An expanded access implementation model (model 2) placed platforms at 74 facilities to cover 77% of HIV-exposed infants, with 23% of tests sent to centralised laboratories (SOC). The third implementation model, termed a hub-and-spoke model, used the same facilities and number of platforms as the first model (hubs), while about 40% of HIV-exposed children provided dried blood spots at the lower throughput sites (spokes) for transport to the hubs. Compared with the base-case model, ICERs for the expanded JHU implementation model were 2–66 times higher, and 1·2–6·6 times higher for the hub-and-spoke model, comparing POC with SOC.^21^ By contrast, CEPAC model analyses were based only on a hub-and-spoke model, as implemented by the EGPAF and Unitaid in Zimbabwe, with two key differences from the Zambian JHU hub-and-spoke model. First, the CEPAC model assumed cost integration (POC platform use 60–90%) compared with the 100% use of the base-case JHU POC model. Second, in the Zimbabwean hub-and-spoke model, spokes were within 1 h travel from hub sites, to enable transport of rapid dried blood spots. The turnaround time for spoke site test results was shorter for the Zimbabwean than the Zambian model (7 days compared with 4 weeks).^19^, ^21^ In all analyses, variations in cost per test (reflecting capital and recurrent costs) affected overall ICER estimates (appendix pp 15–36).^21^, ^22^ Longer time spent on POC testing (partly reflecting staff skill and training) increased the recurrent costs and, therefore, ICER in the same model (appendix pp 15–36). However, in CEPAC model 1, POC remained cost-effective compared with SOC across a plausible range of cost per test (base-case $27·61; range $10–50 in 2016 US$).^18^ Shared use of platforms (both HIV viral load and tuberculosis diagnostics for GeneXpert and HIV viral load only for m-PIMA) was assumed for all SOC scenarios, as is standard for most centralised laboratories. Shared use of platforms in a POC setting substantially improved ICER estimates. Both JHU models evaluated two POC payment approaches: 100% of the capital and recurrent costs associated with POC paid by EID programmes (no shared use), versus POC platform sharing with other services (tuberculosis diagnostics and POC HIV viral load monitoring), with EID programmes paying pro rata by percentage of use.^20^, ^21^ Shared use reduced the overall POC costing substantially, such that POC became cost-saving compared with SOC (table 3, appendix pp 15–36).

## Discussion

Across all available cost-effectiveness mathematical models, POC-NAT-based infant diagnosis of HIV increased treatment coverage and improved survival of children living with HIV compared with SOC, facilitated by earlier diagnosis and timely initiation of ART.^18^, ^19^, ^20^, ^21^ Without shared use of POC platforms, the POC testing strategy might require greater overall expenditure than SOC, but ICERs for all base-case scenarios and outcomes were within acceptable ranges across different clinical outcomes, geographical settings, and testing approaches. These data therefore strongly support appropriately implemented POC as a cost-effective strategy to improve the timely diagnosis and health of children living with HIV.

Moreover, the shorter time horizon models (JHU models 1 and 2) provided compelling evidence that POC could be cost-saving compared with SOC, given shared platform use and cost.^20^, ^21^ This is a key finding of the review, with substantial policy implications. In support of this, a costing study for POC-based infant diagnosis of HIV in Zimbabwe showed that the most significant drivers of cost relate to materials and supplies.^24^ Nonetheless, ongoing collection and review of programmatic costing and effectiveness data should remain a priority as countries scale up their national infant HIV diagnostic programmes, with broader implementation of POC platforms.

Scaling up of POC infant HIV diagnostics might also be affordable in most high HIV burden settings. In the context of the Zimbabwean HIV care and prevention budget, moving from SOC to POC increased spending by an estimated 0·44% of the overall HIV budget and linked 167% more children living with HIV to care over 5 years (CEPAC model 2).^19^ That is, for every 100 children living with HIV initiating ART following SOC-NAT, more than 260 children living with HIV could initiate ART following POC, with less than 1% increased spending in the overall budget. Encouragingly, similar budgetary impact results were noted in JHU model 1.^20^

A broad range of sensitivity analyses across reports highlighted some important considerations around POC implementation. Cost-effectiveness was maximised with targeted implementation in settings with poor SOC performance and low prevention of vertical transmission coverage, provided that results have rapid turnaround times with prompt ART initiation and retention in care for optimal treatment effectiveness. Increasing capital and recurrent costs—as might result from reduced lifespan of POC instruments and increased test run times—will negatively affect cost-effectiveness, emphasising the importance of POC maintenance, staff training and support, and uninterrupted supplies.

These models share the known limitations of mathematical models, including the requisite simplification of complex processes with reliance on assumptions and data from multiple sources.^15^ However, the structure and processes of infant testing were modelled explicitly and reported transparently, using data from high-quality sources for both costing and clinical outcomes.^18^, ^19^, ^20^, ^21^ Despite multiple differences, the model findings are congruent. Nonetheless, the absence of long-term clinical data following POC testing is an important limitation, and programmatic data are urgently needed to this end.^18^, ^19^, ^20^, ^21^ By design, cost-effectiveness models from a health-care perspective do not reflect societal or patient perspectives on potential costs and gains. POC infant testing has high reported maternal acceptability, an important consideration for policy makers seeking patient-centred interventions.^33^, ^34^, ^35^, ^36^, ^37^ Although we used an extensive search strategy, our review is limited to only four reports from two models, all focused on Africa; generalisability is a concern. Nonetheless, the highest incidence and burden of HIV is in sub-Saharan Africa, which is well represented in these models.^7^ We focused on cost-effectiveness models and did not include costing analyses from clinical trials.^38^ Although there are inherent limitations to mathematical models, this approach enables simultaneous comparison of different strategies and approaches beyond the timelines of available data. These cost-effectiveness analyses support the findings of published costing analyses.^17^, ^24^ Direct comparison of ICERs between models is complicated by differences in denomination of the primary outcomes ($ per diagnosis *vs* $ per YLS). There is no established cost-effectiveness threshold for the former outcome, and debate exists regarding the use of Gross Decimal Product for cost-effectiveness thresholds in general.^39^, ^40^ Nonetheless, where an intervention (here, POC testing as modelled in JHU model 2 with shared platform use) is both more effective and less expensive than the alternative (here, SOC testing), concerns around cost-effectiveness thresholds do not apply.

In conclusion, the use of POC-NAT for infant HIV diagnosis is a cost-effective—and potentially cost-saving—strategy compared with laboratory-based NAT, with significant benefits for children living with HIV. However, considered implementation of POC testing platforms, with adequate support and maintenance strategies for both testing and treatment initiation, are required to minimise costs and optimise outcomes. In 2020, the estimated ART coverage for children younger than 15 years was only 54% globally, compared with 74% among adults.^41^ Implementation of POC infant diagnosis is a first step towards addressing this equity gap in HIV treatment coverage, alongside strong antiretroviral treatment programmes for both mothers and children.

## Data sharing

All data used in this manuscript are freely available in the published reports included for the synthesis.

## Declaration of interests

We declare no competing interests.
