# Supplementary material for: Cost-effectiveness of point-of-care versus centralised, laboratory-based nucleic acid testing for diagnosis of HIV in infants: a systematic review of modelling studies
Source: Lancet HIV. 2023 May 4;10(5):e320–31. doi: 10.1016/S2352-3018(23)00029-2 (PMC10175481; doi:10.1016/S2352-3018(23)00029-2)
Supplement: Supplementary appendix [file mmc1.pdf]

THE LANCET
HIV

Supplementary appendix

This appendix formed part of the original submission and has been peer reviewed. We post it as supplied by the authors.

Supplement to: le Roux SM, Odayar J, Sutcliffe CG, et al. Cost-effectiveness of point-of-care versus centralized, laboratory-based nucleic acid testing for diagnosis of HIV in infants: a systematic review of modelling studies. *Lancet HIV* 2023; **10:** e320–31

Cost-effectiveness of point-of-care versus centralized, laboratory-based nucleic acid testing for infant diagnosis of HIV: a systematic overview and narrative synthesis of mathematical modelling studies

Stanzi M le Roux et al

APPENDIX

SUPPLEMENTAL TABLE 1. Search strategy for Pubmed/MEDLINE

#1 HIV	(("hiv"[MeSH Terms] OR "hiv"[All Fields]) OR HIV-exposed[All Fields] OR "HIV exposed"[All Fields])
#2 Early infant diagnosis	#2 Early infant diagnosis<br>((("early infant diagnosis"[All Fields] OR EID[All Fields]) OR<br>(("child"[MeSH Terms] OR "child"[All Fields]) OR<br>("child"[MeSH Terms] OR "child"[All Fields] OR "children"[All Fields]) OR<br>("infant"[MeSH Terms] OR "infant"[All Fields]) OR<br>("infant"[MeSH Terms] OR "infant"[All Fields] OR "infants"[All Fields]) OR<br>("pediatrics"[MeSH Terms] OR "pediatrics"[All Fields] OR "paediatric"[All Fields]) OR ("pediatrics"[MeSH Terms] OR "pediatrics"[All Fields] OR "pediatric"[All Fields])))
#3 Point of care	(rapid[All Fields] OR RDT[All Fields] OR "near-patient"[All Fields] OR POC[All Fields] OR<br>("point-of-care systems"[MeSH Terms] OR<br>("point-of-care"[All Fields] AND "systems"[All Fields]) OR<br>"point-of-care systems"[All Fields] OR<br>("point"[All Fields] AND "care"[All Fields]) OR<br>"point of care"[All Fields]) OR<br>"point of care"[All Fields])
#4 Cost-effectiveness	(("cost-benefit analysis"[MeSH Terms] OR<br>("cost-benefit"[All Fields] AND "analysis"[All Fields]) OR<br>"cost-benefit analysis"[All Fields] OR<br>("cost"[All Fields] AND "effectiveness"[All Fields]) OR "cost effectiveness"[All Fields]) OR<br>"cost effectiveness"[All Fields] OR cost-effective[All Fields] OR "cost effective"[All Fields] OR ICER[All Fields] OR DALY[All Fields]))
#5 Mathematical modelling studies	("Models, Theoretical"[Mesh] OR model[All Fields] OR<br>("Model Driven Eng Lang Syst"[Journal] OR "models"[All Fields]) OR modelling[All Fields] OR<br>("projection"[MeSH Terms] OR "projection"[All Fields] OR "forecasting"[MeSH Terms] OR "forecasting"[All Fields])))
# 6 Combined search	#1 AND #2 AND #3 AND #4 AND #5

Supplemental table 2A. CHEERS checklist for 2 reports of CEPAC-Pediatric model: (i) Frank et al, Lancet HIV 2019; (ii) McCann et al, JAIDS 2020

Section/item	Item nr	Recommendation	(i) Reported on page nr / line nr	(ii) Reported on page nr / line nr
**Title and abstract**				
Title	1	Identify the study as an economic evaluation or use more specific terms such as "cost-effectiveness analysis", and describe the interventions compared	Yes	Yes
Abstract	2	Provide a structured summary of objectives, perspective, setting, methods (including study design and inputs), results (including base case and uncertainty analyses), and conclusions.	Yes, page e182	Yes, page s12
**Introduction**				
Background and objectives	3	Provide an explicit statement of the broader context of the study.<br>Present the study question and its relevance for health policy or practice decisions	Yes, page e182 and e183 under "Introduction"	Yes, pages s12 and s13, under "Introduction"
**Methods**				
Target population and subgroups	4	Describe characteristics of the base case population and subgroups analysed, including why they were chosen.	Yes, page e183 and e184 under "Study design and overview", and "Modelled population and early infant diagnosis strategies"	Yes, page s13 second column under "Analytic Overview"
Setting and location	5	State relevant aspects of the system(s) in which the decision(s) need(s) to be made	Yes, page e183 and e184 under "Study design and overview", and "Modelled population and early infant diagnosis strategies"	Yes, page s13 second column under "Analytic Overview" and page s14 under "Modeled Population and Strategies"
Study perspective	6	Describe the perspective of the study and related this to the costs being evaluated	Yes, page e183 and e184 under "Study design and overview", and "Modelled population and early infant diagnosis strategies"	Yes, page s13 second column under "Analytic Overview"
Comparators	7	Describe the interventions or strategies being compared and state why they were chosen.	Yes, page e184 under "Modelled population and early infant diagnosis strategies"	Yes, page s13 second column under "Analytic Overview" and page s14 under "Modeled Population and Strategies"
Time horizon	8	State the time horizon(s) over which costs and consequences are being evaluated and say why appropriate	Yes, page e183 under "Study design and overview"	Yes, page s13 second column under "Analytic Overview" for overall model (life-time perspective) and also page s16 for five-year perspective
Discount rate	9	Report the choice of discount rate(s) used for costs and outcomes and say why appropriate	Yes, reported (page e184 under "Modelled population and early infant diagnosis strategies) but not specifically justified in this report	Yes, page s13 second column under "Analytic Overview" (not specifically justified in this report)

Section/item	Item nr	Recommendation	(i)<br>Reported on page nr / line nr	(ii)<br>Reported on page nr / line nr
Choice of health outcomes	10	Describe what outcomes were used as the measure(s) of benefit in the evaluation and their relevance for the type of analysis performed.	Yes, page e183 under "Study design and overview"	Yes, page s13 second column under "Analytic Overview"
Measurement of effectiveness	11a	Single study-based estimates: Describe fully the design features of the single effectiveness study and why the single study was a sufficient source of clinical effectiveness data.	Yes, table 1 on page e184 lists multiple studies used as data sources for measures of effectiveness	Yes, table 1 on page s15 lists multiple studies used as data sources for measures of effectiveness
Measurement and valuation of preference-based outcomes	12	If applicable, describe the population and methods used to select preferences for outcomes.	Not applicable	Not applicable
Estimating resources and costs	13b	Model-based economic evaluation: describe approaches and data sources used to estimate resource use associated with model health states. Describe primary or secondary research methods for valuing each resource item in terms of its unit cost. Describe any adjustments made to approximate opportunity costs.	Yes, reported on pages e184 and e185 under "Data sources"<br><br>Opportunity costs not addressed	Yes, page s13 under "Analytic Overview", pages s14-15 under "Data sources"<br><br>Opportunity costs considered in terms of impact on 5-year budget in representative country (page s16)
Currency, price date and conversion	14	Report the dates of the estimated resource quantities and unit costs. Describe methods for adjusting estimated unit costs to year of reported costs if necessary. Describe methods for converting costs into a common currency base and the exchange rate.	Yes, reported on pages e184 and e185 under "Data sources"	Yes, pages s14 and s15, under "Test costs"
Choice of model	15	Describe and give reasons for the specific type of decision-analytical model used. Providing a figure to show model structure is strongly recommended.	Yes, reported on pages e183 and e184, under "Model description"; figure and additional details referenced to appendix, website, and previous publications	Yes, page s13 under "Model structure", with additional details referenced to appendix, website, and previous publications
Assumptions	16	Describe all structural or other assumptions underpinning the decision-analytic mode.	Yes, reported on pages e184 and e185, under "Modelled population and early infant diagnosis strategies", "Data sources" and table 1	Yes, reported pages s13, s14 and s15 under different aspects of methods section; shown in table 1 on page s15
Analytical methods	17	Describe all analytical methods supporting the evaluation. This could include methods for dealing with skewed, missing, or censored data; extrapolation methods; methods for pooling data; approaches to validate or made adjustments (such as half-cycle corrections) to a model; and methods for handling population heterogeneity and uncertainty.	Yes, reported on page e185 under "Scenario and sensitivity analyses"; more details in the appendix	Yes, reported on page s15-16 under "Sensitivity analyses"
**Results**				

Section/item	Item nr	Recommendation	(i) Reported on page nr / line nr	(ii) Reported on page nr / line nr
Study parameters	18	Report the values, ranges, references, and, if used, probability distributions for all parameters. Report reasons or sources for distributions used to represent uncertainty where appropriate. Providing a table to show the input values is strongly recommended.	Yes, reported under methods pages e185 to e186), and table 1	Yes, pages s16 ("Base Case Results: Clinical Outcomes" and "Base Case Results: Cost- and cost-effectiveness outcomes", also Table 2 on page s16 and figure 1
Incremental costs and outcomes	19	For each intervention, report mean values for the main categories of estimated costs and outcomes of interest, as well as mean differences between the comparator groups. If applicable, report incremental cost-effectiveness ratios.	Yes, shown in table 2 (page e185), figure 1 and table 3 (both on page e186), alongside details under "Results" heading, page e187	Yes, pages s16 ("Base Case Results: Clinical Outcomes" and "Base Case Results: Cost- and cost-effectiveness outcomes", also Table 2 on page s16<br>5-year outcomes shown on pages s18 and s19
Characterising uncertainty	20b	Model-based economic evaluation: describe the effects on the results of uncertainty for all input parameters, and uncertainty related to the structure of the model and assumptions.	Yes, described in last paragraph of "Results" on page e186, with Tornado diagram in figure 2 on page e187; also demonstrated in figure 3 (page e188) and table 4 (page e189)	Yes, pages s17 to s18, under scenario and sensitivity analyses sub-headings; also shown in table 3 (page s17) and figure 2 (page s18)
Characterising heterogeneity	21	If applicable, report differences in costs, outcomes, or cost-effectiveness that can be explained by variations between subgroups of patients with different baseline characteristics or other observed variability in effects that are not reducibl3e by more information.	Yes, included in results relating to scenario and sensitivity analyses, last half of "Results" section, pages e186 and e187; also demonstrated in figure 3 (page e188) and table 4 (page e189)	Yes, pages s17 to s18, under scenario and sensitivity analyses sub-headings; also shown in table 3 (page s17) and figure 2 (page s18)
**Discussion**				
Study findings, limitations, generalizability, and current knowledge	22	Summarise key study findings and describe how they support the conclusions reached. Discuss limitations and the generalizability of the findings and how the findings fit with current knowledge.	Yes, discussed in depth in Discussion section, pages e187 to e189	Yes, page s19 to s20
**Other**				
Source of funding	23	Describe how the study was funded and the role of the funder in the identification, design, conduct, and reporting of the analysis. Describe other non-monetary sources of support.	Yes, reported on page e186, under "Role of the funding source"	Yes, front page of publication, page s12
Conflicts of interest	24	Describe any potential for conflict of interest of study contributors in accordance with journal policy. In the absence of a journal policy, we recommend authors comply with International Committee of Medical Journal Editors recommendations.	Yes, reported on page e189 under "Declaration of interests"	Yes, front page of publication, page s12

Supplemental table 2B. CHEERS checklist for 2 reports of Johns Hopkins model: (i) Salvatore et al, AIDS 2021; (ii) De Broucker et al, PLoS ONE 2021

Section/item	Item nr	Recommendation	(i) Salvatore Reported on page nr / line nr, plus comments	(ii) De Broucker Reported on page nr / line nr, plus comments
**Title and abstract**				
Title	1	Identify the study as an economic evaluation or use more specific terms such as "cost-effectiveness analysis", and describe the interventions compared	Yes	Yes
Abstract	2	Provide a structured summary of objectives, perspective, setting, methods (including study design and inputs), results (including base case and uncertainty analyses), and conclusions.	Yes, page 1	Yes, page 1
**Introduction**				
Background and objectives	3	Provide an explicit statement of the broader context of the study.<br>Present the study question and its relevance for health policy or practice decisions	Yes, page1	Yes, page 2
**Methods**				
Target population and subgroups	4	Describe characteristics of the base case population and subgroups analysed, including why they were chosen.	Yes, page 2 under "Setting and model structure"	Yes, page 3, under "Overview and setting"
Setting and location	5	State relevant aspects of the system(s) in which the decision(s) need(s) to be made	Yes, page 2 under "Setting and model structure"	Yes, page 3, under "Overview and setting"
Study perspective	6	Describe the perspective of the study and related this to the costs being evaluated	Yes, page 2 under "Economic outcomes"	No, not explicitly stated although implied throughout that the perspective is from the EID programme
Comparators	7	Describe the interventions or strategies being compared and state why they were chosen.	Yes, page 1 under "Introduction" and page 2 under "Setting and Model structure"	Yes, from page 3 ("Testing algorithms and platforms") through page 10 ("Primary and sensitivity analyses")
Time horizon	8	State the time horizon(s) over which costs and consequences are being evaluated and say why appropriate	Yes, costing horizon at 9 months and outcomes through initiation of ART, death or until 18 months of age.	Yes, page 3 under "Overview and setting"
Discount rate	9	Report the choice of discount rate(s) used for costs and outcomes and say why appropriate	n/a	Yes, in supplementary materials but % not specified
Choice of health outcomes	10	Describe what outcomes were used as the measure(s) of benefit in the evaluation and their relevance for the type of analysis performed.	Yes, reported on page 4 under "Epidemiologic outcomes", "Economic outcomes" and "Cost-effectiveness"	Yes, reported under "Overview and setting" on page 3 and "Health outcomes" on page 10

Section/item	Item nr	Recommendation	(i) Salvatore<br>Reported on page nr / line nr, plus comments	(ii) De Broucker<br>Reported on page nr / line nr, plus comments
Measurement of effectiveness	11a	Single study-based estimates: Describe fully the design features of the single effectiveness study and why the single study was a sufficient source of clinical effectiveness data.	Yes, table 1 page 289 shows multiple studies used as data sources for measures of effectiveness	Yes, table 1 page 9 shows multiple studies used as data sources for measures of effectiveness
Measurement and valuation of preference-based outcomes	12	If applicable, describe the population and methods used to select preferences for outcomes.	n/a	n/a
Estimating resources and costs	13b	Model-based economic evaluation: describe approaches and data sources used to estimate resource use associated with model health states. Describe primary or secondary research methods for valuing each resource item in terms of its unit cost. Describe any adjustments made to approximate opportunity costs.	Yes, reported on page 4 under “Economic outcomes” with detail provided in appendix	Yes, provided in methods section under “Costs” on page 10, with detailed information in appendix
Currency, price date and conversion	14	Report the dates of the estimated resource quantities and unit costs. Describe methods for adjusting estimated unit costs to year of reported costs if necessary. Describe methods for converting costs into a common currency base and the exchange rate.	Yes, reported on page 4 under “Economic outcomes” with detail provided in appendix	Yes, provided in methods section under “Costs” on page 10, with detailed information in appendix
Choice of model	15	Describe and give reasons for the specific type of decision-analytical model used. Providing a figure to show model structure is strongly recommended.	Although choice of model is not justified, figures are provided to explain model structure in the appendix	Although choice of model is not justified, figures are provided to explain model structure in the appendix
Assumptions	16	Describe all structural or other assumptions underpinning the decision-analytic mode.	Yes, described in all sub-sections of the methods, along with details in table 1; additional information available in appendix	Yes, discussed under “Model parameters and data collection” on pages 8, with parameters shown in summary in table 1 and in detail in the appendix
Analytical methods	17	Describe all analytical methods supporting the evaluation. This could include methods for dealing with skewed, missing, or censored data; extrapolation methods; methods for pooling data; approaches to validate or made adjustments (such as half-cycle corrections) to a model; and methods for handling population heterogeneity and uncertainty.	Yes, described under outcome measures generally, and under “Measures of Uncertainty” and “Sensitivity analyses” specifically	Yes, provided on pages 10-11 under “Primary and sensitivity analyses”
**Results**				
Study parameters	18	Report the values, ranges, references, and, if used, probability distributions for all parameters. Report reasons or sources for distributions used to represent uncertainty where appropriate. Providing a table to show the input values is strongly recommended.	Yes, provided in table 1 with additional detail given in the appendix	Yes, provided in table 1 with additional detail given in the appendix

Section/item	Item nr	Recommendation	(i) Salvatore<br>Reported on page nr / line nr, plus comments	(ii) De Broucker<br>Reported on page nr / line nr, plus comments
Incremental costs and outcomes	19	For each intervention, report mean values for the main categories of estimated costs and outcomes of interest, as well as mean differences between the comparator groups. If applicable, report incremental cost-effectiveness ratios.	Yes, reported under "Results" section pages 4-5, along with figures 1 to 3 and additional detail in appendix	Yes, reported under "Comparison of testing algorithms in the primary implementation model" on page 11, and under "Comparison of implementation models" on page 12
Characterising uncertainty	20b	Model-based economic evaluation: describe the effects on the results of uncertainty for all input parameters, and uncertainty related to the structure of the model and assumptions.	Yes, provided in "Results" section under "Sensitivity analyses" on page 5, and illustrated with figure 4	Yes, provided throughout the results section on pages 11-12, with several tables provided in the appendix
Characterising heterogeneity	21	If applicable, report differences in costs, outcomes, or cost-effectiveness that can be explained by variations between subgroups of patients with different baseline characteristics or other observed variability in effects that are not reducibl3e by more information.	Yes, provided in "Results" section under "Sensitivity analyses" on page 5, and illustrated with figure 4	Yes, provided throughout the results section on pages 11-12, with several tables provided in the appendix
**Discussion**				
Study findings, limitations, generalizability, and current knowledge	22	Summarise key study findings and describe how they support the conclusions reached. Discuss limitations and the generalizability of the findings and how the findings fit with current knowledge.	Yes, discussed in detail on pages 6-9	Yes, discussed in detail on pages 12-14
**Other**				
Source of funding	23	Describe how the study was funded and the role of the funder in the identification, design, conduct, and reporting of the analysis. Describe other non-monetary sources of support.	Yes, provided on title page	Yes, provided under "Acknowledgements" on page 14
Conflicts of interest	24	Describe any potential for conflict of interest of study contributors in accordance with journal policy. In the absence of a journal policy, we recommend authors comply with International Committee of Medical Journal Editors recommendations.	Yes, provided on page 9	Yes, on title page

Supplemental table 3. PRISMA checklist

Section/topic	#	Checklist item	Reported on page #
**TITLE**			
Title	1	Identify the report as a systematic review, meta-analysis, or both.	Title page
**ABSTRACT**			
Structured summary	2	Provide a structured summary including, as applicable: background; objectives; data sources; study eligibility criteria, participants, and interventions; study appraisal and synthesis methods; results; limitations; conclusions and implications of key findings; systematic review registration number.	Page 2, abstract
**INTRODUCTION**			
Rationale	3	Describe the rationale for the review in the context of what is already known.	Page 3
Objectives	4	Provide an explicit statement of questions being addressed with reference to participants, interventions, comparisons, outcomes, and study design (PICOS).	Page 3
**METHODS**			
Protocol and registration	5	Indicate if a review protocol exists, if and where it can be accessed (e.g., Web address), and, if available, provide registration information including registration number.	Page 4
Eligibility criteria	6	Specify study characteristics (e.g., PICOS, length of follow-up) and report characteristics (e.g., years considered, language, publication status) used as criteria for eligibility, giving rationale.	Page 4
Information sources	7	Describe all information sources (e.g., databases with dates of coverage, contact with study authors to identify additional studies) in the search and date last searched.	Page 4
Search	8	Present full electronic search strategy for at least one database, including any limits used, such that it could be repeated.	Appendix
Study selection	9	State the process for selecting studies (i.e., screening, eligibility, included in systematic review, and, if applicable, included in the meta-analysis).	Page 4 & 5; supplemental figure 1
Data collection process	10	Describe method of data extraction from reports (e.g., piloted forms, independently, in duplicate) and any processes for obtaining and confirming data from investigators.	Page 4 & 5
Data items	11	List and define all variables for which data were sought (e.g., PICOS, funding sources) and any assumptions and simplifications made.	Page 5
Risk of bias in individual studies	12	Describe methods used for assessing risk of bias of individual studies (including specification of whether this was done at the study or outcome level), and how this information is to be used in any data synthesis.	Page 4

Summary measures	13	State the principal summary measures (e.g., risk ratio, difference in means).	
Synthesis of results	14	Describe the methods of handling data and combining results of studies, if done, including measures of consistency (e.g., I^2) for each meta-analysis.	Not applicable

SUPPLEMENTAL TABLE 4. Details of excluded full-text article

Journal [Year]	First author	Title	Reason(s) for exclusion
The Lancet HIV [2019][1]	Flavia Bianchi	Evaluation of a routine point-of-care intervention for early infant diagnosis of HIV: an observational study in eight African countries	1. Primary outcome: Although costing is provided per test returned, there are no measures of incremental cost-effectiveness<br>2. Study design: observational, not a mathematical modelling study

Supplemental table 5. Detailed overview of model similarities and differences

	CEPAC model #1<br>Zimbabwe	CEPAC model #2<br>Zimbabwe	JHU model #1<br>Sub-Saharan Africa	JHU model #2<br>Zambia
	Value [range examined in sensitivity analysis] or additional information	Value [range examined in sensitivity analysis] or additional information	Value (95% uncertainty range) or additional information	Value [range examined in sensitivity analysis] or additional information
**Setting**				
Country	Zimbabwe	Zimbabwe	"Representative high burden setting in sub-Saharan Africa where POC is likely to be considered"	Zambia
EID testing strategy	6 weeks	6 weeks	6 weeks and 9 months	Birth, 6 weeks, and 6 months
Publication details	Frank et al, Lancet HIV 2019[2]	McCann et al, JAIDS 2020[3]	Salvatore et al, AIDS 2021[4]	De Broucker et al, PLoS ONE 2021[5]
**Model overview**				
Type	Individual-level, state-transition, micro-simulation	Individual-level, state-transition, micro-simulation	Decision tree model	Decision tree model
Cycles	Monthly	Monthly	n/a	n/a
Discount	3% (life expectancy and cost)	3% (life expectancy and costs)	Undiscounted	"Based on lifespan of vehicle/platform and the time horizon (5 years)"
Perspective	Health system	Health system	Health system: EID programme specifically	Health system: EID programme specifically
Time horizon: costs	Birth to death	1. Birth to death<br>2. 5 years	9-month costs	5-year costs
Time horizon: clinical outcomes	Birth to death	1. Birth to death<br>2. 5 years	Excess mortality over 18 months among HIV-infected infants who went undiagnosed or initiated ART more than 60 days after specimen collection	Excess mortality from time of HIV infection to ART start or 12 months of age if ART not started
ICER	Cost per year of life saved (YLS)	Cost per YLS	Cost per additional infant initiating ART within 60 days	
Pre-determined ICER threshold	1x Zimbabwe GDP/ person (2016 USD): ≤ $1010/YLS	Cost per YLS equivalent to providing 2nd line ART:<br>< $580/YLS (2017)<br><br>Also compared to:<br>GDP/person Zimbabwe (2017) $1600/YLS	Not explicitly stated<br>Additional costs of POC over SOC expressed as % of per-capita health expenditure in select countries	Not explicitly stated
Conversion of monetary units to 2018 across models	100 USD$ in 2016<br>= ~105 US$ in 2018	100 USD$ in 2017<br>= ~$102 US$ in 2018	Publication presents findings in 2018 USD	Publication presents findings in 2018 USD

	CEPAC model #1<br>Zimbabwe	CEPAC model #2<br>Zimbabwe	JHU model #1<br>Sub-Saharan Africa	JHU model #2<br>Zambia
	**Value [range examined in sensitivity analysis] or additional information**	**Value [range examined in sensitivity analysis] or additional information**	**Value (95% uncertainty range) or additional information**	**Value [range examined in sensitivity analysis] or additional information**
	(4.68% cumulative inflation, CPI calculator)	(2.49% cumulative inflation, CPI calculator)		
2018 equivalent GDP/person for setting in US$, World Bank WDI	$1684	$1684	$1589	$1556
Primary comparison	POC vs SOC<br><br>*POC*:<br>1 platform<br>1 testing algorithm<br>1 implementation model	POC vs strengthened SOC vs SOC<br><br>*POC*:<br>2 platforms<br>1 testing algorithm<br>1 implementation model	POC vs SOC<br><br>*POC*:<br>3 platforms<br>2 testing algorithms	POC vs SOC<br><br>*POC*:<br>2 platforms<br>4 testing algorithms<br>3 implementation models
Primary outcomes	1. Survival at 12 weeks<br>2. Survival at 12 months<br>3. Life expectancy<br>4. Life-time costs<br>5. ICER per year of life saved	1. Survival at 12 months<br>2. Life expectancy<br>3. Life-time costs<br>4. ICER per year of life saved<br>5. Budget impact over 5 years	1. % initiated on ART by 60 days<br>2. % initiated ART by 18 months<br>3. Excess 18-month mortality prior to ART initiation	1. % initiated on ART by 60 days<br>2. % initiated ART by 12 months<br>3. Excess 12-month mortality prior to ART initiation<br>4. % children treated with ART but false HIV positive test<br>5. ICER per additional outcome (1)-(3)
Sensitivity analyses: one-way	1. % test result return<br>2. Time to test result return<br>3. % ART initiation<br>4. POC and SOC assay characteristics (sensitivity, specificity, and cost)<br>5. MTCT risk<br>6. Duration breastfeeding<br>7. Coverage of ART	1. % test result return<br>2. Time to test result return<br>3. % ART initiation<br>4. Assay characteristics (sensitivity, specificity, and cost)<br>5. Duration breastfeeding<br>6. ART efficacy<br>7. ART costs<br>8. HIV clinical care costs	All model parameters examined; most influential were:<br>1. Capital and recurrent costs<br>2. % ART initiation within 60 days on SOC<br>3. Sensitivity of POC assays<br>4. Background PMTCT coverage	Restricted to POC3 testing and primary implementation model<br>1. Background PMTCT coverage<br>2. % ART initiation for SOC<br>3. Distribution of infants entering testing schedule at different ages<br>4. Retention of infants in testing schedule<br>5. POC platform characteristics<br>- Sensitivity<br>- Lifespan<br>- Utilization rates<br>- Time per test run<br>6. Staff time spent on POC testing

	CEPAC model #1<br>Zimbabwe	CEPAC model #2<br>Zimbabwe	JHU model #1<br>Sub-Saharan Africa	JHU model #2<br>Zambia
	**Value [range examined in sensitivity analysis] or additional information**	**Value [range examined in sensitivity analysis] or additional information**	**Value (95% uncertainty range) or additional information**	**Value [range examined in sensitivity analysis] or additional information**
Sensitivity analyses: multi-way	Combinations of above parameters	Combination of above parameters including finding threshold for equivalence comparing strengthened lab vs POC	Two-way sensitivity analysis incorporating parameters identified as above	For each PMTCT coverage setting, to obtain a worst (all parameters set at worst value) and best (all set to best value) case scenario for POC vs SOC
Scenario analyses	1. Optimistic, intermediate, and pessimistic conditions of uptake along the cascade for both strategies<br>2. Prioritized POC testing for women without ART in pregnancy (SOC for others)<br>3. Worse ART outcomes among POC<br>4. Worse ART outcomes among both POC and SOC	1. Abbott RDxm-PIMA Reagent Rental	1. Integrated POC utilization scenario: machines shared with viral load monitoring ± TB diagnostics<br>2. Combined scenario: POC/SOC=80%/20% (80% of infants access POC; 20% in more remote areas continue with SOC)	1. PMTCT coverage: "Best-case" – 99% constant; baseline, 93% (1% annual increase through 97% in year 5); "worst-case" – 73% (3% annual increase through 85% in year 5)
**Data sources for key model inputs (base case and sensitivity analyses)**				
HIV disease-related				
PMTCT coverage	UNAIDS data for Zimbabwe (2017)	UNAIDS data for Zimbabwe (2018)<br>Zimbabwe Ministry of Health Global AIDS response progress report (2018)	WHO PMTCT estimates by region (2017)	WHO PMTCT estimates by country (2018)
MTCT transmission risks (Infant "HIV prevalence" at testing)	Multiple clinical trials and cohort studies[6-15]	Stover et al, Spectrum/AIM Model for Estimating Key Indicators at National and Sub-national Levels, AIDS (2019)[16]	Multiple clinical trials and cohort studies[6-15]	Multiple clinical trials and cohort studies[6-15]
Maternal CD4 and viral load measures and changes over time, child sex distributions, and other infant characteristics including breastfeeding duration	Francke et al, JID (2016)[17]<br>Zimbabwe National Statistics Agency (2015)	Francke et al, JID (2016)[17]<br>Zimbabwe Ministry of Health programmatic data<br>Dinh et al, CID (2018)[18]	Not modelled	Not modelled
HIV care costs per month	Zimbabwe National AIDS spending assessment, UNAIDS (2012)	Clinton Health Access Initiative (2009)[19]	Not modelled	Not modelled

	CEPAC model #1 Zimbabwe	CEPAC model #2 Zimbabwe	JHU model #1 Sub-Saharan Africa	JHU model #2 Zambia
	**Value [range examined in sensitivity analysis] or additional information**	**Value [range examined in sensitivity analysis] or additional information**	**Value (95% uncertainty range) or additional information**	**Value [range examined in sensitivity analysis] or additional information**
		Global Fund to Fight AIDS, Tuberculosis and Malaria (procurement tool, 2019)[20]		
Cost of ART	Clinton Health Access Initiative (2016)[19]<br>Doherty et al (2014)[21]	Clinton Health Access Initiative (2017)[19]	Not modelled	Not modelled
ART efficacy for children with HIV	Violari et al, NEJM (2012)[22]<br>Palombi et al, NEJM (2010)[8]<br>Walmsley et al, NEJM (2013)[23]	Violari et al, NEJM (2012)[22]<br>Palombi et al, NEJM (2010)[8]<br>Walmsley et al, NEJM (2013)[23]	Not modelled	Not modelled
Mortality risks children	Treated and untreated children, varying by age:<br>Becquet et al, PLoS One (2012)[15]<br>Marston et al, Int J Epi (2011)[24]<br>United Nations, World Population Prospects (2008)	Treated and untreated children, varying by age:<br>Becquet et al, PLoS One (2012)[15]<br>Marston et al, Int J Epi (2011)[24]<br>United Nations, World Population Prospects (2009)	Only for untreated children, varying by age - Newell et al, Lancet (2004)[25]	Only for untreated children, varying by age - Newell et al, Lancet (2004)[25]
Test and platform characteristics				
SOC sensitivity and specificity, error rates	Mallampati et al; Systematic Review, JAIDS (2017)[26]	Mallampati et al; Systematic Review, JAIDS (2017)[26]	Assumption 100% for optimal ("conservative") comparison	Assumption 100% for optimal ("conservative") comparison
POC sensitivity and specificity, error rates	Hsiao et al, PloS ONE (2016)[27]	Hsiao et al, PloS ONE (2016)[27]	WHO Information note on HIV diagnostics (2017)[28]	WHO Information note on HIV diagnostics (2017)[28]
Utilization rates of SOC	Programmatic data from Zimbabwe pilot study, EGPAF/UNITAID EID initiative[1]	Programmatic data from Zimbabwe pilot study, EGPAF/UNITAID EID initiative[1]	CHAI data from 7 countries[19]	Assumption 15%, tested range 10-100%
Utilization rates of POC	Programmatic data from Zimbabwe pilot study, EGPAF/UNITAID EID initiative[1]	Programmatic data from Zimbabwe pilot study, EGPAF/UNITAID EID initiative[1]	NSEBA study[29-31]<br>Ndlovu et al, PLoS One (2018)[32]	NSEBA study[29-31]<br>Ndlovu et al, PLoS One (2018)[32]
Costing for EID				
Cost per test, SOC	Global Fund (2017)[20]<br>Creek et al (2008)[33]	Programmatic data, EGPAF/UNITAID EID project, published in Mukherjee et al, JAIDS (2019)[34]<br>Nichols et al, JAIDS (2019)[35]	Clinton Health Access Initiative[19]	NSEBA study[29-31]<br>Clinton Health Access Initiative[19]
Cost per test, POC	Global Fund (2017)[20]<br>Creek et al (2008)[33]	Programmatic data, EGPAF/UNITAID EID project, published in Mukherjee et al, JAIDS (2019)[34]	Clinton Health Access Initiative[19]	NSEBA study[29-31]<br>Clinton Health Access Initiative[19]

	CEPAC model #1<br>Zimbabwe	CEPAC model #2<br>Zimbabwe	JHU model #1<br>Sub-Saharan Africa	JHU model #2<br>Zambia
	**Value [range examined in sensitivity analysis] or additional information**	**Value [range examined in sensitivity analysis] or additional information**	**Value (95% uncertainty range) or additional information**	**Value [range examined in sensitivity analysis] or additional information**
		Nichols et al, JAIDS (2019)[35]		
EID cascade parameters				
Number of children being tested per time point evaluated	Assumed 100%, to test full impact potential impacts of POC vs SOC	Assumed 100%, to test full impact potential impacts of POC vs SOC	NSEBA study[29-31]<br>Dinh et al, CID (2018)[18]	NSEBA study[29-31]:<br>Sutcliffe et al, BMC Pediatrics (2017)[36]
Probability of receiving EID results, POC & SOC	Bianchi et al Lancet HIV (2018)[1] programmatic data, EGPAF/UNITAID EID initiative	Bianchi et al Lancet HIV (2018)[1] programmatic data, EGPAF/UNITAID EID initiative	Not modelled explicitly	Not modelled explicitly
Turn-around time for first and confirmatory test results, POC & SOC	Bianchi et al Lancet HIV (2018)[1] programmatic data, EGPAF/UNITAID EID initiative	Bianchi et al Lancet HIV (2018)[1] programmatic data, EGPAF/UNITAID EID initiative	Not modelled explicitly	Not modelled explicitly
Probability of linking to care/initiating ART after positive test result, POC & SOC	Bianchi et al Lancet HIV (2018)[1] programmatic data, EGPAF/UNITAID EID initiative	Bianchi et al Lancet HIV (2018)[1] programmatic data, EGPAF/UNITAID EID initiative	NSEBA study[29-31]	NSEBA study[29-31]<br>Sutcliffe et al, BMC Pediatrics (2017)[36]
**Modelled cohort characteristics**				
Number of HIV-exposed children simulated for base-case scenarios	30 million to 300 million	30 million	25 000 (2 tests per child annually, ~50 000 tests)<br>10 000 parameter sets for 95% uncertainty ranges<br>100 000 new parameter sets for sensitivity and threshold analyses	37 500<br>(annual cohort of 7500 children, over 5 years)
Infant mean age, months (sd)	0 (0)	0 (0)	Birth cohort	Children enter the testing cohort at different ages – see below
Infant sex, female/male (%)	51.2%/48.8%	51.2%/48.8%	Not specified	Not specified
Infant CD4% at infection	45% (10)	45% (10)	Not specified	Not specified
Mothers with ≤ 350 cells/uL before ART (%)	36%	36%	Not specified	Not specified
Mothers receiving ART during pregnancy and breastfeeding (%)	93%	96%	“High coverage”: median 93% (84-100)<br>“Low coverage”: median 48% (43-53)	93% [73-99] in primary analysis

	CEPAC model #1<br>Zimbabwe	CEPAC model #2<br>Zimbabwe	JHU model #1<br>Sub-Saharan Africa	JHU model #2<br>Zambia
	**Value [range examined in sensitivity analysis] or additional information**	**Value [range examined in sensitivity analysis] or additional information**	**Value (95% uncertainty range) or additional information**	**Value [range examined in sensitivity analysis] or additional information**
Breastfeeding, % of all mother-infant pairs	80% [0-100]	94%	Not specified	Not specified
Exclusively breastfed 1st 6 months (%)	55% [0-100]	65%	Not specified	Not specified
Mixed feeding for 1st 6 months (%)	25% [0-100]	29%	Not specified	Not specified
Replacement feeding from birth (%)	20% [0-100]	6%	Not specified	Not specified
Mean duration of breastfeeding, months (SD)	18 (2) [6-24]	17 (1) [6-24]	Not specified	Not specified
Uptake of testing				
Proportion testing at birth	n/a	n/a	Not modelled	0.40 [0.15-0.80] of all children enter the cohort at birth
Proportion tested at 6 weeks	1.0	1.0	0.85 (0.83-0.87)	0.45 [0.45-0.10] of all children enter the cohort at 6 weeks
Proportion tested at 6/9 months	n/a	n/a	Returning at 9 months:<br>0.8 (0.7-0.9) of those who tested negative at 6 weeks	0.15 [0.40-0.10] of all children enter the cohort at 6 months
Proportion returning for subsequent testing	n/a	n/a	Returning at 9 months:<br>0.8 (0.7-0.9) of those who tested negative at 6 weeks	Returning for any repeat EID testing after birth:<br>*Received PMTCT:*<br>0.8 [0.75-0.85]<br>*Did not receive PMTCT:*<br>0.6 [0.35-0.85]
Proportion returning after 1 week for tiebreaker (primary POC comparison)	n/a	n/a	0.95 (0.93-0.97)	0.97
Transmission risk overview				
All transmission routes	5.2% (overall, model output)	3.0% (overall, model output)	Among infants first tested at 6 weeks, model input:<br>*Had PMTCT*: 2% (0.5-4%)<br>*No PMTCT*: 20% (10-30%)	Overall, model output: 3.9%<br>Model input, depending on age and previous testing<br>*Received PMTCT*: 1-8%<br>*No PMTCT*: 2-30%
Intra-uterine/intra-partum (once-off)	On ART: 1%	On ART: 0.26-1.4%, depending on when ART started	n/a	Testing at birth:<br>*Received PMTCT*: 1%

	CEPAC model #1<br>Zimbabwe	CEPAC model #2<br>Zimbabwe	JHU model #1<br>Sub-Saharan Africa	JHU model #2<br>Zambia
	**Value [range examined in sensitivity analysis] or additional information**	**Value [range examined in sensitivity analysis] or additional information**	**Value (95% uncertainty range) or additional information**	**Value [range examined in sensitivity analysis] or additional information**
	Not on ART: 17-27% depending on maternal CD4	Not on ART, 18-19.7% depending on chronic or acute HIV		*No PMTCT*: 8%<br>Testing at 6 weeks:<br>Had negative birth testing<br>*Received PMTCT*: 1%<br>*No PMTCT*: 2%<br>Did not have birth testing:<br>*Received PMTCT*: 2%<br>*No PMTCT*: 22%
Postpartum	Monthly, while breastfeeding:<br><br>On ART: 0.19%<br>Not on ART: 0.24% to 1.28% depending on maternal CD4 and exclusivity of breastfeeding	Monthly, while breastfeeding:<br><br>On ART:0.11%<br>Not on ART: 0.89%	New transmissions between 6 weeks and 9 months: 1.7% (0.5-3%)<br><br>Transmission risk at age 9 months (time of test)<br>Among infants first tested at 9 months (ie would include all peripartum transmissions), model input<br>*Received PMTCT*:<br>9% (4-14%)<br>*No PMTCT*:<br>30% (15-45%)	Tested at 6 months:<br>Previously tested negative at 6 weeks:<br>*Received PMTCT*: 1%<br>*No PMTCT:* 1%<br>Had negative test at 6 weeks but no birth test:<br>*Received PMTCT*: 1%<br>*No PMTCT*: 2%<br>Had no previous testing:<br>*Received PMTCT:* 8%<br>*No PMTCT:* 30%
Duration of transmission risk in model	Throughout breastfeeding (up to 2 years): if diagnosis missed at 6 weeks or transmission occurred later, diagnosis presumed to occur at intercurrent severe clinical event or at 18-month visit	Throughout breastfeeding (up to 2 years): if diagnosis missed at 6 weeks or transmission occurred later, diagnosis presumed to occur at intercurrent severe clinical event or at 18-month visit	Until 9 months of age	Until 6 months of age
**Mortality and morbidity inputs**				
Maternal mortality (monthly), %	0.10-0.18% depending on maternal CD4	Not reported	n/a	n/a
Untreated HIV+ children: CD4% decline (monthly, range by age and transmission type)	0.05-4.0%<br>(3-6 CD4 cells/uL range by HIV RNA for > 5 years of age)	0.05-4.0%<br>(3-6 CD4 cells/uL range by HIV RNA for > 5 years of age)	n/a	n/a
HIV+ child: clinical events (monthly, range by type of	0.0-11.6	0.0-11.6	n/a	n/a

	CEPAC model #1<br>Zimbabwe	CEPAC model #2<br>Zimbabwe	JHU model #1<br>Sub-Saharan Africa	JHU model #2<br>Zambia
	Value [range examined in sensitivity analysis] or additional information	Value [range examined in sensitivity analysis] or additional information	Value (95% uncertainty range) or additional information	Value [range examined in sensitivity analysis] or additional information
event, child age and CD4%/CD4), %				
HIV+ child: estimated overall mortality risks (untreated HIV; range by timing of infection, age and duration of risk)	n/a	n/a	RANGE: 0.11-0.44<br><br>Infected by 6 weeks:<br>• Between 6 weeks and 9 months: 0.23 (0.18-0.28)<br>• Between 6 weeks and 18 months: 0.39 (0.34-0.44)<br><br>Infected after 6 weeks and lost to follow up: between 6 weeks and 18 months: 0.30 (0.25-0.35)<br><br>Between 9 and 18 months (infected by 9 months):<br>0.16 (0.11-0.21)	RANGE: 0.012-0.33<br><br>Infected by birth<br>• Birth to 6 weeks: 0.012<br>• Birth to 6 months: 0.18<br>• Birth to 12 months: 0.33<br>Infected at 6 weeks<br>• 6 weeks-6 months: 0.17<br>• 6 weeks-12 months: 0.32<br>Infected at 6 months<br>• 6 to 12 months, among infants first tested at 6 months: 0.15<br>• Between infection and age 12 months, among those uninfected at 6 weeks but infected before 6 months: 0.19
HIV+ child: acute risk of death within 30 days of acute event (range by type of event and age), %	0.4-20.0	0.4-20.0	Not examined	Not examined
HIV+ child: chronic mortality risk (monthly)				
HIV-related death (range by age, CD4%/CD4, and history of prior OI), %	0.1-40.8	0.1-40.8	Not examined	Not examined
Non-AIDS related mortality (range by age and sex), %	0.01-1.60	0.01-1.60	Not examined	Not examined
HIV-exposed uninfected (breastfed) mortality (monthly, range by age), %	0.1-1.0	Not reported	Not examined	Not examined
Paediatric ART input	Provided in separate table: Includes ranges for efficacy, CD4 gain, probability of virological	Provided in separate table: Includes ranges for efficacy, CD4 gain, probability of virological failure,	Not examined	Not incorporated: modelled timelines through ART initiation or,

	CEPAC model #1<br>Zimbabwe	CEPAC model #2<br>Zimbabwe	JHU model #1<br>Sub-Saharan Africa	JHU model #2<br>Zambia
	**Value [range examined in sensitivity analysis] or additional information**	**Value [range examined in sensitivity analysis] or additional information**	**Value (95% uncertainty range) or additional information**	**Value [range examined in sensitivity analysis] or additional information**
	failure, relative risk reduction for opportunistic infections and mortality; monthly loss to follow-up	relative risk reduction for opportunistic infections and mortality; monthly loss to follow-up		if no ART started, until 12 months of age
ART efficacy (HIV RNA <50c/mL at 24 weeks on ART)	1st line ART efficacy 82-91% depending on age	1st line ART efficacy 82-91% depending on age [90-96]	Not specified	Not specified
**EID service approaches**				
Modelled testing ages for EID	6 weeks	6 weeks	6 weeks and 9 months	Birth, 6 weeks, 6 months
SOC (laboratory)				
Transport of specimens	Not specified	Weekly sample transport	Not specified, but variable delays imply variable delivery schedules	Not specified, but variable delays imply variable delivery schedules
Platform	Not specified	Not specified	"Representative", eg Abbott m2000 and Roche CAP/CTM	Roche CAP/CTM
Mechanism of confirmatory testing	Not specified	Not specified	Sent on day of receipt of primary test result, to centralized lab	Sent on day of receipt of primary test result, to centralized lab
Mechanism of tie-breaker testing	Not specified	Not specified	Sent on day of receipt of secondary test result, to centralized lab	Sent on day of receipt of secondary test result, to centralized lab
Estimated number of PCR instruments required	Not specified	Not specified	5 (4-6) PCR instruments (assuming shared utilization and idle time)	3 platforms (3 laboratories)
Utilization	Not specified	1300 tests/platform/year (shared with viral load monitoring, % not specified), over 3 years	50% (45-55%)	0.15 [0.10-1.00]
Time spent to run each test	Not specified	Not specified	Not specified	0.35 hour
Time spent per test on sample collection (includes pre-test counselling)	Not specified	Not specified	Not specified	0.6 hour [0.5-1.00]
Lifespan of platform	Not specified	3 years	Not specified	60 months (5 years)
Strengthened SOC (laboratory)				
Transport of specimens	n/a	Daily sample transport	n/a	n/a
Platform	n/a	Not specified	n/a	n/a
Mechanism of confirmatory testing	n/a	Not specified	n/a	n/a
Mechanism of tie-breaker testing	n/a	Not specified	n/a	n/a
POC (primary comparison)				

	CEPAC model #1<br>Zimbabwe	CEPAC model #2<br>Zimbabwe	JHU model #1<br>Sub-Saharan Africa	JHU model #2<br>Zambia
	**Value [range examined in sensitivity analysis] or additional information**	**Value [range examined in sensitivity analysis] or additional information**	**Value (95% uncertainty range) or additional information**	**Value [range examined in sensitivity analysis] or additional information**
Platform	Not specified	GeneXpert Gel	1. GeneXpert IV (GXpert-IV)<br>2. GeneXpert-Edge (GX-Edge)<br>3. Abbott m-PIMA	1. GeneXpert IV<br>2. Abbott m-PIMA
Primary algorithm and implementation model (including transport of specimens)	Not specified	Hub-and-spoke model (46% spoke)<br><br>100% POC for all HE infants<br><br>DBS transported within 1 hour from spoke sites with same day result	*Primary comparison (POC3)* – primary and confirmatory testing same-day POC, tiebreaker also POC, 1 week later<br><br>Did not explicitly compare further details on implementation of up-scaling	*Primary comparison* (*POC3*) – primary and confirmatory testing same-day POC, tiebreaker also POC, 1 week later<br><br>*Primary implementation model*: 40 POC platforms at 40 high-volume facilities (1.5 EID tests per week), 100% testing by POC, all 7500 children annually; 61% of children testing onsite, 39% referred in person from other facilities [assumes no additional programmatic costs per referral, and no LTFU due to referral process]
Mechanism of confirmatory testing (primary comparison)	Not specified	Same-day, POC	Same-day, POC	Same-day, POC
Mechanism of tie-breaker testing	Not specified	Not specified	One week later, POC	One week later, POC
Estimated number of PCR instruments required	Not specified	Not specified	Primary algorithm: 150 (140-160)<br>POC +SOC: 120 POC plus 3 SOC	40
Utilization	Not specified	Not specified	Primary algorithm - POC 100%<br>POC+SOC - POC 100%, SOC 2.5%<br>POC:SOC/80:20<br>- SOC utilization 17%<br>- POC utilization 100%	Primary analysis [integrated costing]: 1.0 [0.10-1.0] both platforms
Time spent to run each test	Not specified	Not specified	Not specified	0.25 hour [0.17-0.5] both platforms
Time spent per test on sample collection	Not specified	Not specified	Not specified	0.6 hour [0.5-1.0] both platforms
Lifespan of platform	Not specified	Not specified	Not specified	7 years GeneXpert [3 years]<br>5 years m-PIMA [2 years]

	CEPAC model #1<br>Zimbabwe	CEPAC model #2<br>Zimbabwe	JHU model #1<br>Sub-Saharan Africa	JHU model #2<br>Zambia
	**Value [range examined in sensitivity analysis] or additional information**	**Value [range examined in sensitivity analysis] or additional information**	**Value (95% uncertainty range) or additional information**	**Value [range examined in sensitivity analysis] or additional information**
Alternative POC platforms, testing algorithms or implementation model options	None	m-PIMA reagent rental (scenario analysis) – same testing algorithm and implementation model	*1 Alternative testing algorithm: "POC+SOC" testing algorithm:*<br>Primary and confirmatory testing done as POC; DBS tiebreaker sent to SOC lab (47-57% LTFU); 140-160 POC devices and 1 SOC with utilization 2.3-2.7%	*Two "POC+SOC" testing algorithms:*<br>(a) Primary and confirmatory testing done as POC; DBS tiebreaker sent to SOC lab (POC2+SOC)<br>(b) Primary testing POC; confirmatory and tiebreaker DBS testing sent to SOC lab (POC+SOC)
			1 alternative implementation model: "POC:SOC, 80:20"<br>80% of testing done at POC (either all POC or POC+SOC for tiebreakers), while 20% of tests get sent to SOC	*2 alternative implementation models:*<br>(a) *"Expanded access":* 74 platforms at 74 facilities (minimum average 3.5 samples per month), covering 77% of HE population; 23% sent to 3 central laboratories, SOC<br>*(b)* *"Hub-and-spoke":* same 40 platforms at 40 facilities as for primary model [covering 61% HE directly] – but 39% of HE get DBS sent to the primary facilities ("near-POC") with a 4-week return of result expectation (rather than same-day)
			"Integrated utilization":<br>GeneXpert (both): EID 15% as also used for HIV VL and TB testing<br>m-PIMA: EID 25% as also used for HIV VL but not for TB testing	*"Integrated utilization":*<br>GeneXpert: EID 10%<br>m-PIMA: EID 15%
**EID cascade parameters**				
EID uptake (%)	100% [30-100] at 6 weeks	100% at 6 weeks	85% at 6 weeks	95% by 6 weeks
Probability of receiving 1st test results				

	CEPAC model #1<br>Zimbabwe	CEPAC model #2<br>Zimbabwe	JHU model #1<br>Sub-Saharan Africa	JHU model #2<br>Zambia
	**Value [range examined in sensitivity analysis] or additional information**	**Value [range examined in sensitivity analysis] or additional information**	**Value (95% uncertainty range) or additional information**	**Value [range examined in sensitivity analysis] or additional information**
SOC (laboratory)	80% [70-100]	79%	Not explicitly modelled but reflected in probability of starting ART before 60 days	Not explicitly modelled but reflected in probability of starting ART before 60 days
Strengthened SOC (laboratory)	n/a	91% [83-98]		
POC (primary comparison)	99% [40-100]	98%		
Mean delay (sd) between primary test and receipt of results				
SOC (laboratory)	2 (0) [1-4] months	61 days	Not explicitly modelled but reflected in probability of starting ART before 60 days	Not explicitly modelled but reflected in probability of starting ART before 60 days
Strengthened SOC (laboratory)	n/a	53 days [5-60]		
POC (primary comparison)	0 (0) [0-1] months	1 day		
Mean delay (sd) between confirmatory test and receipt of results, months				
SOC (laboratory)	0 (1)	Delayed, no details	Delayed, no details	Delayed, no details
Strengthened SOC (laboratory)	n/a	Delayed, no details	n/a	n/a
POC (primary comparison)	0 (0)	Same day	Same day	Same day
Probability of initiating ART after testing positive (model input)				
SOC (laboratory), %	51.9% [50-100]	52%	<60 days: 20% (13-27%)<br><18 months: 48% (43-53%)	<60 days: 30% [13-42%]<br><12 months: 55% [35-65%]
Strengthened SOC (laboratory), %	n/a	71% [66-86]	n/a	n/a
POC (primary comparison), %	98.5% [40-100]	86%	<60 days: 89% (85-94%)<br><18 months: 95% (85-100%)	<60 days: 90%<br><12 months: 94%
**Conventional (SOC) assay characteristics**				
Sensitivity				
Overall (for all infections)	100% [90-100]	100%	100%	100%
Intrauterine infection (all ages)	100% [90-100]	100%	Not specified	Not specified
Intrapartum infection (month 1)	0	0	Not specified	Not specified

	CEPAC model #1<br>Zimbabwe	CEPAC model #2<br>Zimbabwe	JHU model #1<br>Sub-Saharan Africa	JHU model #2<br>Zambia
	**Value [range examined in sensitivity analysis] or additional information**	**Value [range examined in sensitivity analysis] or additional information**	**Value (95% uncertainty range) or additional information**	**Value [range examined in sensitivity analysis] or additional information**
Intrapartum infection (after month 1)	100% [90-100]	100%	Not specified	Not specified
Postpartum infection (month of infection)	0	0	Not specified	Not specified
Postpartum infection (after month of infection)	100% [90-100]	100%	Not specified	Not specified
Specificity (all ages)	99.6% [98.8-100]	99.6%	100%	100%
Error rate, %	1.4% [1.4-12]	3.8%	Not specified	1%
**Point-of-care (POC) characteristics**				
Sensitivity				
Overall (all infections)	96.9% [60-100]	96.9%	96% (93-99) GeneXpert (both)<br>98% (96-100) m-PIMA<br>[Sensitivity analysis: 80%]	96.8% [92.7-98.9] GeneXpert<br>99.0% [96.4-99.9] m-PIMA
Intrauterine infection (all ages)	96.9% [60-100]	96.9%	Not specified	Not specified
Intrapartum infection (month 1)	0	0	Not specified	Not specified
Intrapartum infection (after month 1)	96.9% [60-100]	96.9%	Not specified	Not specified
Postpartum infection (month of infection)	0	0	Not specified	Not specified
Postpartum infection (after month of infection)	96.9% [60-100]	96.9%	Not specified	Not specified
Specificity (all ages)	100% [90-100]	99.9% [90-100]	99.8% (99.7-99.9) GeneXpert (both)<br>99.9% (99.8-99.9) m-PIMA	99.91% GeneXpert<br>99.97% m-PIMA
Error rate, %	6.0% [6-10]	7.8%	Not specified	9% both platforms
**Overview of estimated costs (as per publication)**				
Cost per test	(Incorporates capital cost)	(Incorporates capital cost)	(Recurrent cost only)	Not provided per test
SOC (laboratory)	$24.18 (1.4% error rate)	$18.10	$15 (14-16)	Not provided per test
Strengthened SOC (laboratory)	n/a	$30.47	n/a	Not provided per test
POC (primary comparison)	$27.61 (6.0% error rate)	$30.71, GeneXpert<br>$29.22, m-PIMA	$20 (18-22), GeneXpert IV and Edge<br>$25 (23-27), m-PIMA	Not provided per test

	CEPAC model #1<br>Zimbabwe	CEPAC model #2<br>Zimbabwe	JHU model #1<br>Sub-Saharan Africa	JHU model #2<br>Zambia
	**Value [range examined in sensitivity analysis] or additional information**	**Value [range examined in sensitivity analysis] or additional information**	**Value (95% uncertainty range) or additional information**	**Value [range examined in sensitivity analysis] or additional information**
Capital costs	Included in cost per test	Included in cost per test	Nine-month capital costs per instrument<br><br>SOC platform: ~$35 000<br>GXpert-IV: ~$5 200<br>GX-Edge: ~$1 800<br>m-PIMA: ~$4 800	Based on POC3 testing algorithm, primary implementation model<br><br>SOC: $129 907<br>GeneXpert: $860 857<br>m-PIMA: $801 680
Total programme cost	Included in cost per test	Included in cost per test		
Costs for HIV-specific clinical care				
HIV care per month (range by age, CD5 and CD4 cell count)	$32.75 – 33.69 [0.5x – 3x]	$32.75 – 33.69 [0.5x – 3x]	n/a	n/a
CD4 test	$4.79	$4.79	n/a	n/a
Viral load test	$17.50	$17.50	n/a	n/a
ART regimen per month (range by regimen, dose, and age and weight of infant)	$8.50-44.00 [0.5x – 3x]	$5.49-22.62 [0.5x – 3x]	n/a	n/a
**RESULTS**				
**Clinical outcomes: All HIV-exposed**				
Survival at 12 months, %				
SOC (laboratory)	93.1%	93.5%	n/a	n/a
Strengthened SOC (laboratory)	n/a	93.5%	n/a	n/a
POC (primary comparison)	93.4%	93.7%	n/a	n/a
Total life-expectancy				
SOC (laboratory)	62.5, undiscounted years<br>25.69, discounted years	63.35, undiscounted years<br>25.97, discounted years	n/a	n/a
Strengthened SOC (laboratory)	n/a	63.38, undiscounted years<br>25.99, discounted years	n/a	n/a
POC (primary comparison)	62.6, undiscounted years<br>25.77, discounted years	63.43, undiscounted years<br>26.02, discounted years	n/a	n/a
**Clinical outcomes: CWH**				

	CEPAC model #1<br>Zimbabwe	CEPAC model #2<br>Zimbabwe	JHU model #1<br>Sub-Saharan Africa	JHU model #2<br>Zambia
	**Value [range examined in sensitivity analysis] or additional information**	**Value [range examined in sensitivity analysis] or additional information**	**Value (95% uncertainty range) or additional information**	**Value [range examined in sensitivity analysis] or additional information**
Survival at 12 weeks, %				
SOC (laboratory)	76.1%	Not reported	n/a	n/a
Strengthened SOC (laboratory)	n/a	Not reported	n/a	n/a
POC (primary comparison)	83.5%	Not reported	n/a	n/a
ART initiated by 60 days, %				
SOC (laboratory)	n/a	n/a	*High coverage PMTCT:* 19 %<br>*Low coverage PMTCT:*20%	*Base case*: 27.8%<br>*High coverage PMTCT:* 27.5%<br>*Low coverage PMTCT*: 28.2%
POC (primary comparison)	n/a	n/a	*High coverage PMTCT (93%)*<br>82% GeneXpert both<br>84% m-PIMA<br>*Low coverage PMTCT (48%)*<br>85% GeneXpert both<br>87% m-PIMA	GeneXpert/m-PIMA:<br>*Base case (93%)*<br>81.4% / 82.8%<br>*High coverage PMTCT*<br>80.6% / 82.0%<br>*Low coverage PMTCT*<br>82.7% / 84.1%
Scenario for POC:SOC = 80:20 (only high coverage, EID 100% of capital costs)	n/a	n/a	*High coverage PMTCT (93%)*<br>70% GeneXpert both<br>71% m-PIMA	Not examined
Other POC testing algorithms	n/a	n/a	POC+SOC:<br>*High coverage PMTCT (93%)*<br>80% GeneXpert both<br>83% m-PIMA<br>*Low coverage PMTCT (48%)*<br>83% GeneXpert both<br>85% m-PIMA	GeneXpert/m-PIMA<br>*Base case only (93%)*<br>POC2+SOC: 79.8%/82.2%<br>POC+SOC: 81.5%/82.8%
Survival at 12 months, %				
SOC (laboratory)	69.0%	67.3%	n/a	n/a
Strengthened SOC (laboratory)	-	69.9%	n/a	n/a
POC (primary comparison)	78.0%	75.6%	n/a	n/a
ART initiated by 12 months, %				
SOC (laboratory)	n/a	n/a	n/a	*Base case*: 50.9%<br>*High coverage PMTCT:* 50.4%<br>*Low coverage PMTCT*: 51.8%

	CEPAC model #1<br>Zimbabwe	CEPAC model #2<br>Zimbabwe	JHU model #1<br>Sub-Saharan Africa	JHU model #2<br>Zambia
	**Value [range examined in sensitivity analysis] or additional information**	**Value [range examined in sensitivity analysis] or additional information**	**Value (95% uncertainty range) or additional information**	**Value [range examined in sensitivity analysis] or additional information**
POC (primary comparison)	n/a	n/a	n/a	GeneXpert/m-PIMA:<br>*Base case (93%)*<br>85.0% / 86.4%<br>*High coverage PMTCT*<br>84.2% / 85.6%<br>*Low coverage PMTCT*<br>86.4% / 87.8%
Other POC testing algorithms	n/a	n/a	n/a	GeneXpert/m-PIMA:<br>*Base case only*<br>POC2+SOC: 84.0%/86.1%<br>POC+SOC: 85.1%/86.5%
ART initiated by 18 months, %				
SOC (laboratory)	n/a	n/a	*High coverage PMTCT:* 46%<br>*Low coverage PMTCT:* 47%	n/a
POC (primary comparison)	n/a	n/a	*High coverage PMTCT (93%)*<br>87% GeneXpert both<br>89% m-PIMA<br>*Low coverage PMTCT (48%)*<br>90% GeneXpert both<br>92% m-PIMA	n/a
Scenario for POC:SOC = 80:20 (only high coverage, EID 100% of capital costs)	n/a	n/a	*High coverage PMTCT (93%)*<br>79% GeneXpert both<br>80% m-PIMA	n/a
Other POC testing algorithms	n/a	n/a	POC+SOC:<br>*High coverage PMTCT*<br>86% GeneXpert both<br>88% m-PIMA<br>*Low coverage PMTCT*<br>89% GeneXpert both<br>91% m-PIMA	n/a
HIV-related death by 12/18 months			Death by 18 months	Death by 12 months
SOC (laboratory)	n/a	n/a	*High coverage PMTCT:* 23%<br>*Low coverage PMTCT:* 26%	*Base case:* 18.1%<br>*High coverage PMTCT:* 17.5%<br>*Low coverage PMTCT:* 19.0%
POC (primary comparison)	n/a	n/a	Utilization: 100% EID	GeneXpert/m-PIMA:

	CEPAC model #1<br>Zimbabwe	CEPAC model #2<br>Zimbabwe	JHU model #1<br>Sub-Saharan Africa	JHU model #2<br>Zambia
	**Value [range examined in sensitivity analysis] or additional information**	**Value [range examined in sensitivity analysis] or additional information**	**Value (95% uncertainty range) or additional information**	**Value [range examined in sensitivity analysis] or additional information**
			High coverage PMTCT<br>5% GeneXpert both<br>5% m-PIMA<br>*Low coverage PMTCT*<br>5% GeneXpert both<br>4% m-PIMA	*Base case (93%)*<br>4.2% / 3.8%<br>*High coverage PMTCT*<br>4.2% / 3.9%<br>*Low coverage PMTCT*<br>4.1% / 3.7%
Scenario for POC:SOC = 80:20 (only high coverage, EID 100% of capital costs)	n/a	n/a	POC utilization: 100% EID<br>*High coverage PMTCT*<br>9% GeneXpert both<br>8% m-PIMA	Not examined
Other POC testing algorithms	n/a	n/a	POC+SOC:<br>*High coverage PMTCT*<br>5% GeneXpert both<br>5% m-PIMA<br>*High coverage PMTCT*<br>5% GeneXpert both<br>5% m-PIMA	GeneXpert/m-PIMA:<br>*Base case only:*<br>POC2+SOC: 4.6% / 4.0%<br>POC+SOC: 4.2% / 3.8%
Total life-expectancy, undiscounted years (CWH)				
SOC (laboratory)	22.7 undiscounted years	21.74 undiscounted years	n/a	n/a
Strengthened SOC (laboratory)	n/a	22.71 undiscounted years	n/a	n/a
POC (primary comparison)	25.5 undiscounted years	24.49 undiscounted years	n/a	n/a
**Economic outcomes: details on costing approaches**	"Total cost of ownership" [includes costs of logistics (shipping), reagent/cartridges, equipment (set-up, training, installation, service), maintenance (3 years), controls, calibrations, and consumables<br><br>Costing data obtained from Global Fund's TCO estimates (2017); HIV costs from treatment facilities and Zimbabwe 2012 National AIDS spending assessment; costs for ART,	Assays, materials, supplies, facility updates, labour, training, monitoring, maintenance, shipping, and sample transport<br><br>Costing data obtained from resource utilization study in Zimbabwe, CHAI, and published costing data; EGPAF programmatic data; Kenyan data for	Capital costs: instruments, maintenance, freight, insurance, inspection, handling, clearance/shipping/ distribution, and internet connectivity (annualized over useful lives of 5-7 years per instrument)<br><br>Recurrent costs: reagents, consumables, sample collection, transportation, salaries, and waste management	Capital costs: Platform (purchase, service, maintenance, freight, installation, insurance); Vehicles; Staff training; Communication costs<br><br>Recurrent costs: reagents and supplies for testing, supplies for specimen collection, transportation of samples, salaries for specimen collection, processing, and testing, and waste management.

	CEPAC model #1<br>Zimbabwe	CEPAC model #2<br>Zimbabwe	JHU model #1<br>Sub-Saharan Africa	JHU model #2<br>Zambia
	**Value [range examined in sensitivity analysis] or additional information**	**Value [range examined in sensitivity analysis] or additional information**	**Value (95% uncertainty range) or additional information**	**Value [range examined in sensitivity analysis] or additional information**
	CD4 and VL from Global Fund and CHAI.	strengthened SOC, converted to equivalent costs in Zimbabwe	Costing data obtained primarily from Clinton Health Access Initiative (CHAI)	Costing data obtained from NSEBA study, CHAI and Unicef supply division
**Economic outcomes: HIV-exposed**				
Total cost per test (as per publication)	(Incorporates capital cost)	(Incorporates capital cost)	(Incorporates capital cost)	
SOC (laboratory)	$24.18 (1.4% error rate)	$ 18.10 (3.8% error rate)	*High coverage PMTCT (93%)*<br>$17.08<br>*Low coverage PMTCT*<br>$17.10	Not provided
Strengthened SOC (laboratory)	n/a	$ 30.47 (3.8% error rate)	n/a	Not provided
POC (primary comparison)	$27.61 (6.0% error rate)	$ 30.71 [GeneXpert Gel] (7.8% error rate)<br>$ 29.33 m-PIMA (6.7% error rate)	*High coverage PMTCT (93%)*<br>*EID only:*<br>$38.27 GXpert-IV<br>$26.35 GX-Edge<br>$41.91 m-PIMA<br>*Integrated (cost shared):*<br>$22.76 GXpert-IV<br>$20.95 GX-Edge<br>$29.23 m-PIMA<br><br>*Low coverage PMTCT (48%)*<br>*EID only:*<br>$37.97 GXpert-IV<br>$26.24 GX-Edge<br>$41.64 m-PIMA<br>*Integrated (cost shared):*<br>$22.71 GXpert-IV<br>$20.94 GX-Edge<br>$29.17 m-PIMA	Not provided
POC:SOC = 80:20 (only high coverage, EID 100%	n/a	n/a	*High coverage PMTCT only*<br>*EID only, using 80% POC only:*<br>$34.10 GXpert-IV	n/a

	CEPAC model #1<br>Zimbabwe	CEPAC model #2<br>Zimbabwe	JHU model #1<br>Sub-Saharan Africa	JHU model #2<br>Zambia
	**Value [range examined in sensitivity analysis] or additional information**	**Value [range examined in sensitivity analysis] or additional information**	**Value (95% uncertainty range) or additional information**	**Value [range examined in sensitivity analysis] or additional information**
of capital costs): total cost per POC test			$24.53 GX-Edge<br>$37.03 m-PIMA	
Alternative POC testing algorithms	n/a	n/a	POC+SOC:<br>*High coverage PMTCT (93%)*<br>*EID only:*<br>$38.28 GXpert-IV<br>$26.35 GX-Edge<br>$41.91 m-PIMA<br>*Integrated (cost shared):*<br>$22.76 GXpert-IV<br>$20.96 GX-Edge<br>$29.24 m-PIMA<br><br>*Low coverage PMTCT (48%)*<br>*EID only:*<br>$37.97 GXpert-IV<br>$26.24 GX-Edge<br>$41.63 m-PIMA<br>*Integrated (cost shared):*<br>$22.71 GXpert-IV<br>$20.94 GX-Edge<br>$29.17 m-PIMA	n/a
Lifetime costs: per publication, and [converted to US$2018]				
SOC (laboratory)	$610 [638] undiscounted<br>$370 [387] discounted	$330 [338] undiscounted<br>$200 [205] discounted	n/a	n/a
Strengthened SOC (laboratory)	n/a	$360 [369] undiscounted<br>$220 [225] discounted	n/a	n/a
POC (primary comparison)	$690 [721] undiscounted*<br>$420 [439] discounted	$390 [399] undiscounted<br>$240 [246] discounted	n/a	n/a
**Economic outcomes: CWH**				
Cost per test done	As above	As above	Not presented separately for CWH	Not presented separately for CWH
Lifetime costs: per publication, and [converted to US$2018]				
SOC (laboratory)	$11 830 [12 377]	$10 340 [10 593]	n/a	n/a

	CEPAC model #1<br>Zimbabwe	CEPAC model #2<br>Zimbabwe	JHU model #1<br>Sub-Saharan Africa	JHU model #2<br>Zambia
	**Value [range examined in sensitivity analysis] or additional information**	**Value [range examined in sensitivity analysis] or additional information**	**Value (95% uncertainty range) or additional information**	**Value [range examined in sensitivity analysis] or additional information**
Strengthened SOC (laboratory)	n/a	$10 870 [11 135]	n/a	n/a
POC (primary comparison)	$13 460 [14 083]	$11 800 [12 088]	n/a	n/a
**Incremental cost-effectiveness ratios**				
ICER for ART initiation by 60 days: per additional HIV+ child				
POC vs SOC (US$ 2018) – EID 100%	n/a	n/a	*High coverage PMTCT (93%)*<br>$966 GXpert-IV<br>$430 GX-Edge<br>$1097 m-PIMA<br>*Low coverage PMTCT (48%)*<br>$417 GXpert-IV<br>$191 GX-Edge<br>$475 m-PIMA	*Base case (93% coverage)*<br>$23 GeneXpert<br>$1554 m-PIMA
POC vs SOC (US$ 2018) – EID integrated costs			*High coverage PMTCT (93%)*<br>$268 GXpert-IV<br>$187 GX-Edge<br>$543 m-PIMA<br>*Low coverage PMTCT (48%)*<br>$268 GXpert-IV<br>$187 GX-Edge<br>$543 m-PIMA	*Base case (93% coverage, POC3 and primary implementation model)*<br>GeneXpert: -$831 (cost-saving)<br>m-PIMA: $821
Scenario for POC:SOC = 80:20 (US$ 2018; only high coverage, EID 100% of capital costs, tie-breaker with POC))			*High coverage PMTCT (93%)*<br>$966 GXpert-IV<br>$431 GX-Edge<br>$1097 m-PIMA	n/a
Other POC testing algorithms (100% EID)	n/a	n/a	POC+SOC:<br>*High coverage PMTCT (93%)*<br>$1004 GXpert-IV<br>$447 GX-Edge<br>$1117 m-PIMA<br>*Low coverage PMTCT (48%)*<br>$434 GXpert-IV<br>$198 GX-Edge	GeneXpert/m-PIMA<br>*Base case (93% coverage)*<br>POC2+SOC: $68 / $1609<br>POC+SOC: $73 / $1570

	CEPAC model #1<br>Zimbabwe	CEPAC model #2<br>Zimbabwe	JHU model #1<br>Sub-Saharan Africa	JHU model #2<br>Zambia
	**Value [range examined in sensitivity analysis] or additional information**	**Value [range examined in sensitivity analysis] or additional information**	**Value (95% uncertainty range) or additional information**	**Value [range examined in sensitivity analysis] or additional information**
			$483 m-PIMA	
ICER for <u>ART initiation by 12/18 months</u>: per additional HIV+ child			ART BY 18 MONTHS	ART BY 12 MONTHS
POC vs SOC (US$ 2018) – EID 100%	n/a	n/a	*High coverage PMTCT (93%)*<br>$1467 GXpert-IV<br>$655 GX-Edge<br>$1642 m-PIMA<br>*Low coverage PMTCT (48%)*<br>$633 GXpert-IV<br>$288 GX-Edge<br>$711 m-PIMA	*Base case (93% coverage)*<br>$37 GeneXpert<br>$2406 m-PIMA
POC vs SOC (US$ 2018) – EID integrated costs	n/a	n/a	*High coverage PMTCT (93%)*<br>$406 GXpert-IV<br>$285 GX-Edge<br>$815 m-PIMA<br>*Low coverage PMTCT (48%)*<br>$406 GXpert-IV<br>$285 GX-Edge<br>$815 m-PIMA	*Base case (93% coverage, POC3 and primary implementation model)*<br>GeneXpert: - $1307 (cost-saving)<br>m-PIMA: $1272
Scenario for POC:SOC = 80:20 (US$ 2018; only high coverage, EID 100% of capital costs, tie-breaker with POC))	n/a	n/a	*High coverage PMTCT (93%)*<br>$1468 GXpert-IV<br>$655 GX-Edge<br>$1643 m-PIMA	n/a
Other POC testing algorithms (100% EID)	n/a	n/a	POC+SOC:<br>*High coverage PMTCT (93%)*<br>$1522 GXpert-IV<br>$679 GX-Edge<br>$1670 m-PIMA<br>*Low coverage PMTCT (48%)*<br>$658 GXpert-IV<br>$300 GX-Edge<br>$723 m-PIMA	GeneXpert/m-PIMA<br>*Base case only*<br>POC2+SOC: $106 / $2491<br>POC+SOC: $115 / $2430

	CEPAC model #1<br>Zimbabwe	CEPAC model #2<br>Zimbabwe	JHU model #1<br>Sub-Saharan Africa	JHU model #2<br>Zambia
	**Value [range examined in sensitivity analysis] or additional information**	**Value [range examined in sensitivity analysis] or additional information**	**Value (95% uncertainty range) or additional information**	**Value [range examined in sensitivity analysis] or additional information**
ICER for <u>per death averted</u> prior to ART initiation, HIV+ children:				
POC vs SOC (US$ 2018) – EID 100%	n/a	n/a	*High coverage PMTCT (93%)*<br>$3426 GXpert-IV<br>$1527 GX-Edge<br>$3888 m-PIMA<br>*Low coverage PMTCT (48%)*<br>$1297 GXpert-IV<br>$591 GX-Edge<br>$1475 m-PIMA	*Base case (93% coverage)*<br>$90 GeneXpert<br>$5976 m-PIMA
POC vs SOC (US$ 2018) – EID integrated costs	n/a	n/a	*High coverage PMTCT (93%)*<br>$953 GXpert-IV1<br>$664 GX-Edge<br>$1929 m-PIMA<br>*Low coverage PMTCT (48%)*<br>$953 GXpert-IV<br>$664 GX-Edge<br>$1929 m-PIMA	*Base case (93% coverage, POC3 and primary implementation model)*<br>GeneXpert: -$3199 (cost-saving)<br>m-PIMA: $3159
Scenario for POC:SOC = 80:20 (US$ 2018; only high coverage, EID 100% of capital costs, tie-breaker with POC))	n/a	n/a	*High coverage PMTCT (93%)*<br>$3428 GXpert-IV<br>$1528 GX-Edge<br>$3888 m-PIMA	n/a
Other POC testing algorithms (100% EID)	n/a	n/a	POC+SOC:<br>*High coverage PMTCT (93%)*<br>$3574 GXpert-IV<br>$1593 GX-Edge<br>$3963 m-PIMA<br>*Low coverage PMTCT (48%)*<br>$1357 GXpert-IV<br>$618 GX-Edge<br>$1507 m-PIMA	GeneXpert/m-PIMA<br>*Base case only*<br>POC2+SOC: $211 / $5088<br>POC+SOC: $224 / $4973
ICER per <u>year of life saved: all HIV-exposed</u>				

	CEPAC model #1<br>Zimbabwe	CEPAC model #2<br>Zimbabwe	JHU model #1<br>Sub-Saharan Africa	JHU model #2<br>Zambia
	**Value [range examined in sensitivity analysis] or additional information**	**Value [range examined in sensitivity analysis] or additional information**	**Value (95% uncertainty range) or additional information**	**Value [range examined in sensitivity analysis] or additional information**
POC vs SOC	$680/YLS (~67% annual GDP/person), USD 2016	GeneXpert Gel: $830/YLS (~50% annual GDP/person)<br>m-PIMA: $790/YLS	n/a	n/a
POC vs SOC (US$ 2018)	$711 in 2018	GeneXpert: $850 in 2018	n/a	n/a
Strengthened SOC vs SOC, or vs POC	n/a	Weakly dominated	n/a	n/a
**Affordibility**				
SOC (laboratory)	Not reported	1680 CWH linked to care; $15.7 million total cost; 0.93% of HIV budget over 5 years [Total cost in US$ 2018: $16.1 million]	DRC, Kenya, and Malawi: 0.06%, 0.04% and 0.21% of per capita health expenditure in 2018	Not reported
Strengthened SOC (laboratory)	Not reported	2740 CWH linked to care; $21.6 million total cost; 1.28% of HIV budget over 5 years [Total cost in US$ 2018: $22.1 million]	n/a	Not reported
POC (primary comparison)	Not reported	4480 CWH linked to care; $23.1 million total cost; 1.37% of HIV budget over 5 years [Total cost in US$ 2018: $23.7 million]	EID only (no integration of costs) 0.07% to 0.53% depending on platform and country<br><br>Integrated costs for platforms: 0.06% to 0.37% depending on platform and country	Not reported
**Summary results of sensitivity analyses: thresholds where primary comparison POC no longer cost-effective compared to SOC**				
PMTCT coverage				
High vs low coverage	Not examined	Not examined	Lower ICERS for areas with lower PMTCT coverage	For GeneXpert, ICERs similar across coverage ranges<br>For m-PIMA, ICERs almost doubled for high compared to low coverage for ART<60 days and ART <12 months
Breastfeeding duration	Robust for full range	Robust for full range	Not examined	Not examined

	CEPAC model #1<br>Zimbabwe	CEPAC model #2<br>Zimbabwe	JHU model #1<br>Sub-Saharan Africa	JHU model #2<br>Zambia
	**Value [range examined in sensitivity analysis] or additional information**	**Value [range examined in sensitivity analysis] or additional information**	**Value (95% uncertainty range) or additional information**	**Value [range examined in sensitivity analysis] or additional information**
EID uptake	Robust for full range	Not examined	Not examined	Not examined
SOC assay characteristics and costs				
Sensitivity	Robust for full range	Robust for full range	Not examined	Not examined
Specificity	Robust for full range	Robust for full range	Not examined	Not examined
Cost per test	Robust for full range	If strengthened lab test < $25.71 then more efficient than POC (ICER $740 compared to lab)<br>(~$758 in 2018)	Not examined	Not examined
Health care costs (2016)	ICER>$1010 if >2X higher for both SOC and POC	Robust for full range	Not examined	Not examined
Capital costs	Not examined	Not examined	Not examined	Not examined
Recurrent costs	Not examined	Not examined	Not examined	Not examined
Lifespan of POC machine	Not examined	Not examined	Not examined	Longer SOC platform lifespan increases ICERs slightly for both GeneXpert but not m-PIMA
Utilization %	Not examined	Not examined	Not examined	Not examined
Time to result return	Robust for full range	Robust for full range comparing strengthened lab to standard lab	Greater %ART initiation prior to 60 days for SOC strategy=less cost-effective POC (Threshold > 60%)	Not significantly different whether or not probability of ART initiation increases or decreases after SOC, except for death averted using m-PIMA as POC platform (improved SOC associated with higher ICER)
POC assay characteristics and costs				
Sensitivity	Robust up to < 65%	Robust up to <70% (S-SOC better option for lower POC sensitivity)	ICERS increase as sensitivity decreases: at 80%, ICERs remain favourable as long as % ART initiation < 60 days for POC is higher than for SOC; potentially cost-saving if integrated costs and cost/test < $15	Across tested range, ICERS remain stable (lowest tested sensitivity is 92% for GeneXpert)
Specificity	Robust up to < 92%	Robust for full range; ICER $900 (~$922 in 2018) for specificity of 90%	Robust	Not examined

	CEPAC model #1<br>Zimbabwe	CEPAC model #2<br>Zimbabwe	JHU model #1<br>Sub-Saharan Africa	JHU model #2<br>Zambia
	**Value [range examined in sensitivity analysis] or additional information**	**Value [range examined in sensitivity analysis] or additional information**	**Value (95% uncertainty range) or additional information**	**Value [range examined in sensitivity analysis] or additional information**
Cost per test	Robust up to > $60	Robust up to ≥ $60; if cost ≤ $18 then ICER $520/YLS<br>~$532 in 2018	Robust; in integrated costs scenario, if recurrent cost per POC test < $15, then POC becomes cost-saving	Not directly examined but implicit in sensitivity analyses of varying lifespan, integrated use, and test run time
Health care costs	ICER>$1010 if >2X higher for both SOC and POC	Robust for full range (S-SOC dominated throughout); 0.5 to 3x costs: ICER for POC vs LAB range $640-1630 (~$656-1671 in 2018)	Not examined	Not examined
Capital costs	Not examined	Not examined	See utilization	See utilization
Test run time (sample collection and run)	Not examined	Not examined	Not examined	*Shorter run-time*<br>GeneXpert: cost-saving<br>m-PIMA: similar to base<br><br>*Longer run-time*<br>GeneXpert: Substantially higher<br>m-PIMA: similar to base
Lifespan of POC machine	Not examined	Not examined	Not examined	Shorter lifespan substantially increases ICERs across outcomes and platforms
Utilization %	Not examined	Not examined	Shared capital costs substantially reduce ICERs across platforms and outcomes	GeneXpert (100% to 10%)<br>ART@60d: $23 to -831<br>ART@12m: $37 to -1307<br>Deaths averted: $90 to -3199<br>m-PIMA (100% to 15%)<br>ART@60d: $1554 to 821<br>ART@12m: $2406 to 1272<br>Deaths averted: $5976 to 3159
Probability of test result return				
SOC (laboratory)	Robust for full range	Robust for full range	Not examined	Not examined
Strengthened SOC (laboratory)	n/a	Robust for full range in one-way; mostly robust for multi-way except scenario detailed below	Not examined	Not examined
POC (primary comparison)	Robust up to < 50%	Robust up to ≤60%	Not examined	Not examined

	CEPAC model #1 Zimbabwe	CEPAC model #2 Zimbabwe	JHU model #1 Sub-Saharan Africa	JHU model #2 Zambia
	**Value [range examined in sensitivity analysis] or additional information**	**Value [range examined in sensitivity analysis] or additional information**	**Value (95% uncertainty range) or additional information**	**Value [range examined in sensitivity analysis] or additional information**
Probability of initiating ART			[By 60 days]	
SOC (laboratory)	Robust for full range	Robust for full range	Greater %ART initiation prior to 60 days for SOC strategy=less cost-effective (Threshold > 60%)	See time to result return, above
Strengthened SOC (laboratory)	n/a	Robust for full range	n/a	n/a
POC (primary comparison)	Robust up to <45%	Robust up to ≤ 50%	If lower sensitivity, then fewer children will initiate ART (refer POC sensitivity)	Not examined
Cost of ART	ICER>$1010 if >3X higher for both SOC and POC	Robust for full range (S-SOC dominated throughout); 0.5 to 3x costs: ICER for POC vs LAB range $760-1170 (~$779-1199 in 2018)	Not examined	Not examined
Proportion of children returning for subsequent testing	n/a	n/a	Not examined	ICERS for lower retention:<br>GeneXpert: ~ 2-3x higher<br>m-PIMA: ~ similar to base case<br>ICERS for higher retention:<br>GeneXpert: cost-saving<br>m-PIMA: ~ similar to base case
Timing of infant testing	n/a	n/a	Not examined	If a greater proportion of infants enter the cohort at birth then ICERS improve (becomes marginally cost-saving for GeneXpert)
Multi-way interpretation	See scenario analyses	Robust except*: if S-SOC has same % result return as POC, same %ART initiation as POC and S-SOC TAT<=10 days<br>OR S-SOC cheaper AND %return %ART and return time all equivalent to POC	See scenario analyses: primary drivers for changing cost-effectiveness are<br>• % ART initiation POC vs SOC<br>• Capital and recurrent costs for POC (integrated use substantially improves ICERS)<br>• Sensitivity of POC	See scenario analyses: primary drivers for changing cost-effectiveness (especially GeneXpert) are<br>• POC costs lowered (ICERS improved) by integration or shorter test/run times<br>• POC costs (and ICERS) increased by longer test/run times, and shorter lifespan of POC platform

	CEPAC model #1<br>Zimbabwe	CEPAC model #2<br>Zimbabwe	JHU model #1<br>Sub-Saharan Africa	JHU model #2<br>Zambia
	**Value [range examined in sensitivity analysis] or additional information**	**Value [range examined in sensitivity analysis] or additional information**	**Value (95% uncertainty range) or additional information**	**Value [range examined in sensitivity analysis] or additional information**
**Scenario analyses**				
"Best-worst" case scenarios: ICER in 2016 US$, per year of life saved [~equivalent in 2018 US$)				
POC, optimistic				
vs SOC, optimistic	$650 (~$680 in 2018)	Not examined	Not examined	Not examined
vs SOC, intermediate	$720 (~$754 in 2018)	Not examined	Not examined	Not examined
vs SOC, pessimistic	$750 (~$785 in 2018)	Not examined	Not examined	Not examined
POC, intermediate				
vs SOC, optimistic	Less effective, less expensive	Not examined	Not examined	Not examined
vs SOC, intermediate	$720 (~$754 in 2018)	Not examined	Not examined	Not examined
vs SOC, pessimistic	$760 (~$795 in 2018)	Not examined	Not examined	Not examined
POC, pessimistic				
vs SOC, optimistic	Less effective, less expensive	Not examined	Not examined	Not examined
vs SOC, intermediate	$590 (~$618 in 2018)	Not examined	Not examined	Not examined
vs SOC, pessimistic	$730 (~$764 in 2018)	Not examined	Not examined	Not examined
Best-worst case scenarios: ICER in 2018 US$, per short term outcome, by PMTCT coverage [ICERs for Gene Xpert only]				
ART<60 DAYS				
Primary coverage (93%)	n/a	n/a	n/a	
Primary analysis	n/a	n/a	n/a	$23
Best case	n/a	n/a	n/a	-$717 (cost-saving)
Worst case	n/a	n/a	n/a	$2394
High coverage (99%)				
As per primary analysis	n/a	n/a	n/a	$17
Best case	n/a	n/a	n/a	-$1109 (cost-saving)
Worst case	n/a	n/a	n/a	$3076
Low coverage (77%)				
As per primary analysis	n/a	n/a	n/a	$34
Best case	n/a	n/a	n/a	-$269 (cost-saving)
Worst case	n/a	n/a	n/a	$1223
ART<12 MONTHS				

	CEPAC model #1<br>Zimbabwe	CEPAC model #2<br>Zimbabwe	JHU model #1<br>Sub-Saharan Africa	JHU model #2<br>Zambia
	**Value [range examined in sensitivity analysis] or additional information**	**Value [range examined in sensitivity analysis] or additional information**	**Value (95% uncertainty range) or additional information**	**Value [range examined in sensitivity analysis] or additional information**
Primary coverage (93%)				
Primary analysis	n/a	n/a	n/a	$37
Best case	n/a	n/a	n/a	-$938 (cost-saving)
Worst case	n/a	n/a	n/a	$4269
High coverage (99%)				
As per primary analysis	n/a	n/a	n/a	$27
Best case	n/a	n/a	n/a	-$1452 (cost-saving)
Worst case	n/a	n/a	n/a	$5417
Low coverage (77%)				
As per primary analysis	n/a	n/a	n/a	$54
Best case	n/a	n/a	n/a	-$352 (cost-saving)
Worst case	n/a	n/a	n/a	$2227
DEATH AVERTED				
Primary coverage (93%)				
Primary analysis	n/a	n/a	n/a	$90
Best case	n/a	n/a	n/a	-$2765 (cost-saving)
Worst case	n/a	n/a	n/a	$9201
High coverage (99%)				
As per primary analysis	n/a	n/a	n/a	$67
Best case	n/a	n/a	n/a	-$4211 (cost-saving)
Worst case	n/a	n/a	n/a	$11539
Low coverage (77%)				
As per primary analysis	n/a	n/a	n/a	$126
Best case	n/a	n/a	n/a	-$1269 (cost-saving)
Worst case	n/a	n/a	n/a	$5120
Reduced ART efficacy following POC HIV diagnosis	Robust until < 40%	Not examined	Not examined	Not examined
Reduced ART efficacy following both SOC and POC HIV diagnosis	Robust for full range	Robust for full range	Not examined	Not examined
Reagent rental scenario	Not examined	The strengthened lab approach remains dominated;<br>POC vs SOC: ICER $790 (~$810 in 2018)	Not examined	Not examined

	CEPAC model #1<br>Zimbabwe	CEPAC model #2<br>Zimbabwe	JHU model #1<br>Sub-Saharan Africa	JHU model #2<br>Zambia
	**Value [range examined in sensitivity analysis] or additional information**	**Value [range examined in sensitivity analysis] or additional information**	**Value (95% uncertainty range) or additional information**	**Value [range examined in sensitivity analysis] or additional information**
Alternative priorities/delivery mechanisms				
ICER for "Prioritized POC": SOC	Weakly dominated (ICER $680 (~$712 in 2018))	Not examined	Not examined	Not examined
ICER for POC-SOC (first 2 tests POC, tiebreaker SOC) vs SOC	n/a	n/a	Results similar to POC for all three tests including the tie breaker as indicated	Not examined – sensitivity analyses restricted to POC3 testing algorithm
ICER for "expanded implementation model"	n/a	n/a	n/a	GeneXpert:<br>All outcomes, ICERS ~60x higher<br>m-PIMA:<br>All outcomes, ICERS ~2x higher
ICER for "hub-and-spoke model"	n/a	n/a	n/a	~6 to 7-fold higher ICERS: (GeneXpert only)

Abbreviations: CEPAC Cost-effectiveness of Preventing AIDS Complications, pediatric model; MA, Massachusetts; MD, Maryland; USA, United States of America; JHU, Johns Hopkins University; POC, point-of-care; EID, early infant diagnosis (PCR-based diagnosis in the first 9-18 months of life); YLS, years of life saved; GDP, gross domestic product; ART, antiretroviral therapy; USD, US dollar; SOC, standard-of-care (centralized, laboratory-based testing); S-SOC, strengthened centralized laboratory-based testing; ICER, incremental cost-effectiveness ratio; PMTCT, prevention of mother to child transmission of HIV; n/a, not applicable; PCR, polymerase chain reaction; VL, viral load; CHAI, Clinton Health Access Initiative; CWH, children with HIV

REFERENCES

1. Bianchi F, Cohn J, Sacks E, Bailey R, Lemaire JF, Machekano R. Evaluation of a routine point-of-care intervention for early infant diagnosis of HIV: an observational study in eight African countries. *Lancet HIV* 2019; **6**(6): e373-e81.
2. Frank SC, Cohn J, Dunning L, et al. Clinical effect and cost-effectiveness of incorporation of point-of-care assays into early infant HIV diagnosis programmes in Zimbabwe: a modelling study. *The Lancet HIV* 2019; **6**(3): e182-e90.
3. McCann NC, Cohn J, Flanagan C, et al. Strengthening Existing Laboratory-Based Systems vs. Investing in Point-of-Care Assays for Early Infant Diagnosis of HIV: A Model-Based Cost-Effectiveness Analysis. *J Acquir Immune Defic Syndr* 2020; **84 Suppl 1**(1): S12-s21.
4. Salvatore PP, de Broucker G, Vojnov L, Moss WJ, Dowdy DW, Sutcliffe CG. Modeling the cost-effectiveness of point-of-care platforms for infant diagnosis of HIV in sub-Saharan African countries. *Aids* 2021; **35**(2): 287-97.
5. De Broucker G, Salvatore PP, Mutembo S, et al. The cost-effectiveness of scaling-up rapid point-of-care testing for early infant diagnosis of HIV in southern Zambia. *PloS one* 2021; **16**(3): e0248217.
6. Fawzi W, Msamanga G, Spiegelman D, et al. Transmission of HIV-1 through breastfeeding among women in Dar es Salaam, Tanzania. *J Acquir Immune Defic Syndr* 2002; **31**(3): 331-8.
7. Thior I, Lockman S, Smeaton LM, et al. Breastfeeding plus infant zidovudine prophylaxis for 6 months vs formula feeding plus infant zidovudine for 1 month to reduce mother-to-child HIV transmission in Botswana: a randomized trial: the Mashi Study. *Jama* 2006; **296**(7): 794-805.
8. Palombi L, Marazzi MC, Voetberg A, Magid NA. Treatment acceleration program and the experience of the DREAM program in prevention of mother-to-child transmission of HIV. *AIDS* 2007; **21 Suppl 4**: S65-71.
9. Kilewo C, Karlsson K, Ngarina M, et al. Prevention of mother-to-child transmission of HIV-1 through breastfeeding by treating mothers with triple antiretroviral therapy in Dar es Salaam, Tanzania: the Mitra Plus study. *J Acquir Immune Defic Syndr* 2009; **52**(3): 406-16.
10. Peltier CA, Ndayisaba GF, Lepage P, et al. Breastfeeding with maternal antiretroviral therapy or formula feeding to prevent HIV postnatal mother-to-child transmission in Rwanda. *AIDS* 2009; **23**(18): 2415-23.
11. Shapiro RL, Hughes MD, Ogwu A, et al. Antiretroviral regimens in pregnancy and breast-feeding in Botswana. *The New England journal of medicine* 2010; **362**(24): 2282-94.
12. de Vincenzi I. Triple antiretroviral compared with zidovudine and single-dose nevirapine prophylaxis during pregnancy and breastfeeding for prevention of mother-to-child transmission of HIV-1 (Kesho Bora study): a randomised controlled trial. *Lancet Infect Dis* 2011; **11**(3): 171-80.
13. Taha TE, Li Q, Hoover DR, et al. Postexposure prophylaxis of breastfeeding HIV-exposed infants with antiretroviral drugs to age 14 weeks: updated efficacy results of the PEPI-Malawi trial. *J Acquir Immune Defic Syndr* 2011; **57**(4): 319-25.
14. Thomas TK, Masaba R, Borkowf CB, et al. Triple-antiretroviral prophylaxis to prevent mother-to-child HIV transmission through breastfeeding--the Kisumu Breastfeeding Study, Kenya: a clinical trial. *PLoS Med* 2011; **8**(3): e1001015.
15. Becquet R, Marston M, Dabis F, et al. Children who acquire HIV infection perinatally are at higher risk of early death than those acquiring infection through breastmilk: a meta-analysis. *PloS one* 2012; **7**(2): e28510.
16. Stover J, Glaubius R, Mofenson L, et al. Updates to the Spectrum/AIM model for estimating key HIV indicators at national and subnational levels. *AIDS* 2019; **33 Suppl 3**(Suppl 3): S227-s34.
17. Francke JA, Penazzato M, Hou T, et al. Clinical Impact and Cost-effectiveness of Diagnosing HIV Infection During Early Infancy in South Africa: Test Timing and Frequency. *J Infect Dis* 2016; **214**(9): 1319-28.
18. Dinh TH, Mushavi A, Shiraishi RW, et al. Impact of Timing of Antiretroviral Treatment and Birth Weight on Mother-to-Child Human Immunodeficiency Virus Transmission: Findings From an 18-Month Prospective Cohort of a Nationally Representative Sample of Mother-Infant Pairs During the Transition From Option A to Option B+ in Zimbabwe. *Clinical infectious diseases : an official publication of the Infectious Diseases Society of America* 2018; **66**(4): 576-85.
19. Clinton Health Access Initiative. https://www.clintonhealthaccess.org/ (accessed 8 January 2023).
20. Global Fund to Fight AIDS, Tuberculosis and Malaria. HIV viral load and early infant diagnosis selection and procurement information tool. https://www.theglobalfund.org/en/sourcing-management/health-products/viral-load-early-infant-diagnosis/ (accessed 7 January 2023).
21. Doherty K, Essajee S, Penazzato M, Holmes C, Resch S, Ciaranello A. Estimating age-based antiretroviral therapy costs for HIV-infected children in resource-limited settings based on World Health Organization weight-based dosing recommendations. *BMC Health Serv Res* 2014; **14**: 201.
22. Violari A, Cotton MF, Gibb DM, et al. Early antiretroviral therapy and mortality among HIV-infected infants. *The New England journal of medicine* 2008; **359**(21): 2233-44.
23. Walmsley SL, Antela A, Clumeck N, et al. Dolutegravir plus abacavir-lamivudine for the treatment of HIV-1 infection. *The New England journal of medicine* 2013; **369**(19): 1807-18.

24. Marston M, Becquet R, Zaba B, et al. Net survival of perinatally and postnatally HIV-infected children: a pooled analysis of individual data from sub-Saharan Africa. *Int J Epidemiol* 2011; **40**(2): 385-96.
25. Newell ML, Coovadia H, Cortina-Borja M, Rollins N, Gaillard P, Dabis F. Mortality of infected and uninfected infants born to HIV-infected mothers in Africa: a pooled analysis. *Lancet* 2004; **364**(9441): 1236-43.
26. Mallampati D, Ford N, Hannaford A, Sugandhi N, Penazzato M. Performance of Virological Testing for Early Infant Diagnosis: A Systematic Review. *J Acquir Immune Defic Syndr* 2017; **75**(3): 308-14.
27. Hsiao NY, Dunning L, Kroon M, Myer L. Laboratory Evaluation of the Alere q Point-of-Care System for Early Infant HIV Diagnosis. *PloS one* 2016; **11**(3): e0152672.
28. WHO. Information note - HIV diagnostics: novel point-of-care tools for early infant diagnosis of HIV. Geneva, Switzerland; World Health Organization, 2017. https://www.who.int/publications/i/item/WHO-HIV-2017.16 (accessed 6 January 2023).
29. Jani IV, Meggi B, Loquiha O, et al. Effect of point-of-care early infant diagnosis on antiretroviral therapy initiation and retention of patients. *AIDS* 2018; **32**(11): 1453-63.
30. Mwenda R, Fong Y, Magombo T, et al. Significant Patient Impact Observed Upon Implementation of Point-of-Care Early Infant Diagnosis Technologies in an Observational Study in Malawi. *Clinical infectious diseases : an official publication of the Infectious Diseases Society of America* 2018; **67**(5): 701-7.
31. Sutcliffe CG, Mutanga JN, Moyo N, et al. Acceptability and feasibility of testing for HIV infection at birth and linkage to care in rural and urban Zambia: a cross-sectional study. *BMC Infect Dis* 2020; **20**(1): 227.
32. Ndlovu Z, Fajardo E, Mbofana E, et al. Multidisease testing for HIV and TB using the GeneXpert platform: A feasibility study in rural Zimbabwe. *PloS one* 2018; **13**(3): e0193577.
33. Creek T, Tanuri A, Smith M, et al. Early diagnosis of human immunodeficiency virus in infants using polymerase chain reaction on dried blood spots in Botswana's national program for prevention of mother-to-child transmission. *Pediatr Infect Dis J* 2008; **27**(1): 22-6.
34. Mukherjee S, Cohn J, Ciaranello AL, et al. Estimating the Cost of Point-of-Care Early Infant Diagnosis in a Program Setting: A Case Study Using Abbott m-PIMA and Cepheid GeneXpert IV in Zimbabwe. *J Acquir Immune Defic Syndr* 2020; **84 Suppl 1**: S63-s9.
35. Nichols BE, Girdwood SJ, Crompton T, et al. Monitoring viral load for the last mile: what will it cost? *J Int AIDS Soc* 2019; **22**(9): e25337.
36. Sutcliffe CG, Thuma PE, van Dijk JH, et al. Use of mobile phones and text messaging to decrease the turnaround time for early infant HIV diagnosis and notification in rural Zambia: an observational study. *BMC Pediatr* 2017; **17**(1): 66.
